# Imaging 3D chemistry at 1 nm resolution with fused multi-modal electron tomography

**DOI:** 10.1038/s41467-024-47558-0

**Published:** 2024-04-26

**Authors:** Jonathan Schwartz, Zichao Wendy Di, Yi Jiang, Jason Manassa, Jacob Pietryga, Yiwen Qian, Min Gee Cho, Jonathan L. Rowell, Huihuo Zheng, Richard D. Robinson, Junsi Gu, Alexey Kirilin, Steve Rozeveld, Peter Ercius, Jeffrey A. Fessler, Ting Xu, Mary Scott, Robert Hovden

**Affiliations:** 1 Department of Materials Science and Engineering, University of Michigan, Ann Arbor, MI USA; 2 Mathematics and Computer Science Division, Argonne National Laboratory, Lemont, IL USA; 3 Advanced Photon Source Facility, Argonne National Laboratory, Lemont, IL USA; 4 Department of Material Science and Engineering, Northwestern University, Evanston, IL USA; 5 Department of Materials Science and Engineering, University of California at Berkeley, Berkeley, CA USA; 6 National Center for Electron Microscopy, Molecular Foundry, Lawrence Berkeley National Laboratory, Berkeley, CA USA; 7 Department of Chemistry and Chemical Biology, Cornell University, Ithaca, NY USA; 8 Argonne Leadership Computing Facility, Argonne National Laboratory, Lemont, IL USA; 9 Department of Material Science and Engineering, Cornell University, Ithaca, NY USA; 10 Kavli Institute at Cornell for Nanoscale Science, Cornell University, Ithaca, NY USA; 11 Dow Chemical Co., Collegeville, PA USA; 12 Dow Chemical Co., Terneuzen, the Netherlands; 13 Dow Chemical Co., Midland, MI USA; 14 Department of Electrical Engineering and Computer Science, University of Michigan, Ann Arbor, MI USA; 15 Materials Science Division, Lawrence Berkeley National Laboratory, Berkeley, CA USA; 16 Applied Physics Program, University of Michigan, Ann Arbor, MI USA

**Keywords:** Imaging techniques, Characterization and analytical techniques

## Abstract

Measuring the three-dimensional (3D) distribution of chemistry in nanoscale matter is a longstanding challenge for metrological science. The inelastic scattering events required for 3D chemical imaging are too rare, requiring high beam exposure that destroys the specimen before an experiment is completed. Even larger doses are required to achieve high resolution. Thus, chemical mapping in 3D has been unachievable except at lower resolution with the most radiation-hard materials. Here, high-resolution 3D chemical imaging is achieved near or below one-nanometer resolution in an Au-Fe_3_O_4_ metamaterial within an organic ligand matrix, Co_3_O_4_-Mn_3_O_4_ core-shell nanocrystals, and ZnS-Cu_0.64_S_0.36_ nanomaterial using fused multi-modal electron tomography. Multi-modal data fusion enables high-resolution chemical tomography often with 99% less dose by linking information encoded within both elastic (HAADF) and inelastic (EDX/EELS) signals. We thus demonstrate that sub-nanometer 3D resolution of chemistry is measurable for a broad class of geometrically and compositionally complex materials.

## Abstract (web summary)

The authors demonstrate 3D chemical imaging of organic and inorganic materials near or below one-nanometer resolution using multi-modal electron tomography, by fusing elastic and inelastic scattering signals.

## Introduction

Knowing the complete chemical arrangement of matter in all dimensions is fundamental to engineering novel nanomaterials^1^. Although electron tomography provides comprehensive 3D structure at resolutions below 1 nm using elastic scattering signals^2–4^, chemical tomography obtained from inelastic scattering remains largely out of reach. Several demonstrations of chemical tomography using electron energy loss or x-ray energy spectroscopy (EELS/EDX) accompanied the introduction of scanning transmission electron microscope (STEM) tomography and provide a milestone for 3D imaging^5–8^. However, chemical tomography from core-excitation spectroscopy demands high electron doses that almost always exceed the specimen limits (e.g., >10^7 ^e/Å^2^)^9–11^. If attempting chemical tomography, researchers must sacrifice resolution by collecting few specimen projections (e.g., 5–10) and constrain the total dose (e.g., <10^6 ^e/Å^2^). Consequently, 3D resolution is penalized from undersampling and noisy chemical maps^12^. Therefore, a paradigm shift is necessary for high-resolution chemical tomography.

Achieving high-resolution 3D chemistry at lower dose requires fusing both elastic and inelastic scattering signals. Typically these detector signals are analyzed separately and correlated^13–18^. However, correlative imaging disregards shared but also complementary information between structure and chemistry and misses opportunities to recover useful information^19^. Data fusion, popularized in satellite imaging, goes further than correlation by linking separate signal modalities to reconstruct new information and improve measurement accuracy^20–22^. Early approaches for tomographic data fusion showed noise reduction through joint regularization across modalities^23^ and linear models between EDX and elastic signals^24–26^. Recent developments in 2D multi-modal data fusion integrate the non-linear physics of Rutherford scattering to substantially reduce the dose requirements to acquire an atomic-resolution map^27^. In alignment with this principle of fused multi-modal electron microscopy, we extend its algorithmic framework into the third dimension.

Here we present fused multi-modal electron tomography that offers high-resolution recovery of nanomaterial chemistry in 3D with high signal-to-noise (SNR) by fusing signals from both elastic high-angle annular dark field (HAADF) and inelastic (EDX/EELS) scattering. Multi-modal electron tomography reconstructs the volumetric chemical structure of specimens by solving a three-term inverse problem that fuses information from multiple detectors. This approach extends beyond 2D data fusion^27^ to offer a 3D framework with distinct sampling strategies that minimize dose and maximize resolution. When many HAADF projections are measured alongside far fewer chemical projections 100-fold dose reductions are achievable. Although the 3D chemical structure is severely underdetermined, fusing both modalities allows missing chemical information to become identifiable. This approach demonstrates that researchers can measure 3D chemistry at 1 nm resolution using electron doses as low as 10^4^ e/Å^2^ and as few as nine spectroscopic maps while remaining consistent with original measurements. Multi-modal tomography is validated across multiple material systems, including Au-Fe_3_O_4_ superlattice clusters, core-shell Co_3_O_4_-Mn_3_O_4_^28^, ZnS-Cu_0.64_S_0.36_ heterostructures^29^, Cu-SiC nanoparticles and several simulated specimens. By fusing modalities, chemical tomography is possible at sub-nanometer resolution along all three dimensions is achievable for a wider class of material systems.

## Results

### Principles of fused multi-modal electron tomography

**Fig. 1:**
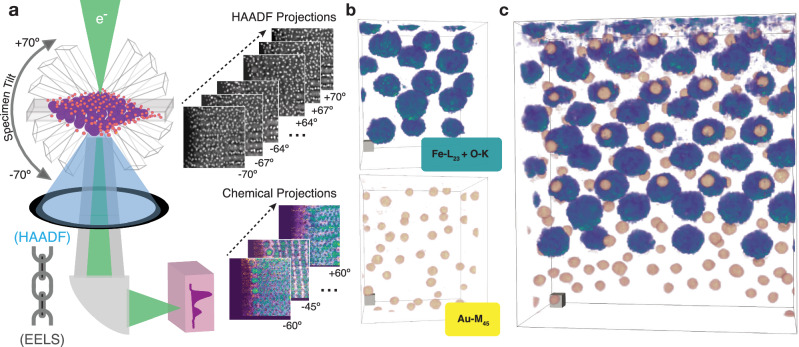
Nanoscale recovery of Au-Fe_3_O_4_ nanoparticle superlattice. **a** Schematic highlighting the linked HAADF and EELS modalities for chemical tomography^84^. HAADF projection images are collected at every tilt increment while core-loss EELS spectra are sparsely acquired every few tilts. **b** The fused multi-modal reconstruction for the specimen’s Fe L_2,3_ (turquoise), O-K (turquoise), and gold M_4,5_ edge (yellow). **c** Chemical overlay of the superlattice nanoparticles over the entire 115 nm field of view. Scale cubes, 5 nm^3^.

High-resolution 3D chemical imaging is achieved using the multi-modal electron tomography framework illustrated in Fig. 1a for a binary Au-Fe_3_O_4_ nanoparticle superlattice grafted with thiol end-functionalized polystyrene ligands. In multi-modal electron tomography, projections of the specimen structure are measured from a HAADF detector and the specimen chemistry is extracted from spectroscopy (EELS or EDX). These two detector modalities are fused during the reconstruction process to provide the complete 3D chemical distribution of a specimen at high resolution and SNR. Figure 1b shows the 3D reconstruction of each individual chemistry: larger 10.2 ± 1.1 nm Fe nanoparticles (blue) and smaller Au 3.9 ± 0.4 nm nanoparticles (orange). Both chemistries are visualized simultaneously in Fig 1c to show the self-organization of the chemical superlattice. The light-element, carbon matrix is shown in Supplementary Fig. 1.

In multi-modal tomography, the number of structural HAADF projections usually exceeds the chemical projections. In this first demonstration, only nine chemical maps (Δ*θ* = 15 °) are measured from the Fe-L_2,3_ and Au-M_4,5_ core-excitation edges in an EELS spectrum whereas 47 HAADF images (Δ*θ* = 3 °) are collected over a ± 70 ° specimen tilt range. Linking both modalities into the reconstruction enables a clear distinction between Fe_3_O_4_ and Au nanoparticles at high resolution from just a few EELS maps and a total electron dose of 5 × 10^5^ e/Å^2^—roughly two orders of magnitude lower total electron dose than an equivalent conventional approach.

Fused multi-modal electron tomography reconstructs three-dimensional chemical models by solving an optimization problem seeking a solution that strongly agrees with (1) the HAADF modality containing high SNR, (2) the chemically sensitive spectroscopic modality (EELS and/or EDX), and (3) encourages sparsity in the gradient domain producing solutions with reduced spatial variation. The overall optimization function is as follows:

1 $$\begin{array}{c} {{{{{{\mathrm{arg}}}}}}\;{{{{{\mathrm{min}}}}}}}_{{{{{{\boldsymbol{x}}}}}}_i \geq 0} \quad \frac{\lambda_1}{2} \left\| {{{{{\boldsymbol{A}}}}}}_h \sum\limits_{i} (Z_i{{{{{\boldsymbol{x}}}}}}_{i})^\gamma - {{{{{\boldsymbol{b}}}}}}_{H} \right\|_2^2+\\ \lambda_2 \sum\limits_{i} \left({{{{{\boldsymbol{1}}}}}}^T {{{{{\boldsymbol{A}}}}}}_c {{{{{\boldsymbol{x}}}}}}_i - {{{{{\boldsymbol{b}}}}}}_{i}^T \log({{{{{\boldsymbol{A}}}}}}_c {{{{{\boldsymbol{x}}}}}}_i+\varepsilon) \right)+\lambda_3 \sum\limits_{i} \|{{{{{\boldsymbol{x}}}}}}_i\|_{{{{{{\mathrm{TV}}}}}}},\end{array}$$

***x***_*i*_ is the reconstructed 3D chemical distributions for element *i*, ***b***_*i*_ is the measured 2D chemical maps for element *i*, ***b***_*H*_ is the measured HAADF micrographs, ***A***_*h*_ and ***A***_*c*_ are forward projection operators for HAADF and chemical modalities, *λ* are regularization parameters, *ε* herein prevents log(0) issues but can also account for background, the $\log$ is applied element-wise to its arguments, superscript *T* denotes vector transpose, and ***1*** denotes the vector of ${N}_{{{{{{{{\rm{chem}}}}}}}}}^{{{{{{{{\rm{proj}}}}}}}}}{n}_{y}{n}_{i}$ ones, where *n*_*y*_ is the number of pixels, *n*_*i*_ is the number of elements present, and ${N}_{{{{{{{{\rm{chem}}}}}}}}}^{{{{{{{{\rm{proj}}}}}}}}}$ is the number of projections for the chemical modality. Pseudo-code for numerical implementation is provided in the Supplementary Materials.

The three terms in Eq. (1) define our fused multi-modal framework designed to surpass traditional limits for chemical tomography. First, we assume a forward model where the simultaneous HAADF is a linear combination of the reconstructed 3D elemental distributions (${{{{{{{{\boldsymbol{x}}}}}}}}}_{i}^{\gamma }$ where *γ* ∈ [1.4, 2]). The incoherent linear imaging approximation for elastic scattering scales with atomic number as ${Z}_{i}^{\gamma }$, where experimentally *γ* is typically around 1.7^30–32^. This *γ* is bounded between 4 and 3 as described by Lenz–Wentzel expressions for electrons passing through a screened coulombic potential and 2 for Rutherford scattering from bare nuclear potentials^33,34^. Second, we ensure the recovered 3D distributions maintain a high degree of data fidelity with the initial measurements by using the log-likelihood for spectroscopic measurements dominated by low-count Poisson statistics^22,35^. In a higher count regime, this term can be substituted with a least-squares discrepancy ($\parallel {{{{{{{\boldsymbol{Ax}}}}}}}}-{{{{{{{\boldsymbol{b}}}}}}}}{\parallel }_{2}^{2}$)^36^. Lastly, we include channel-wise isotropic total variation (TV) regularization to enforce a sparse gradient magnitude, which reduces noise by promoting image smoothness while preserving sharp features^37^. This sparsity constraint, popularized by the field of compressed sensing (CS), is a powerful yet modest prior for recovering structured data^38,39^. When solving Eq. (1), each of these three terms should be weighted appropriately by determining coefficients (*λ*) that balance their contributions. Ultimately, optimization of all three terms is necessary for accurate recovery (Supplementary Figs. 2,3).

**Fig. 2:**
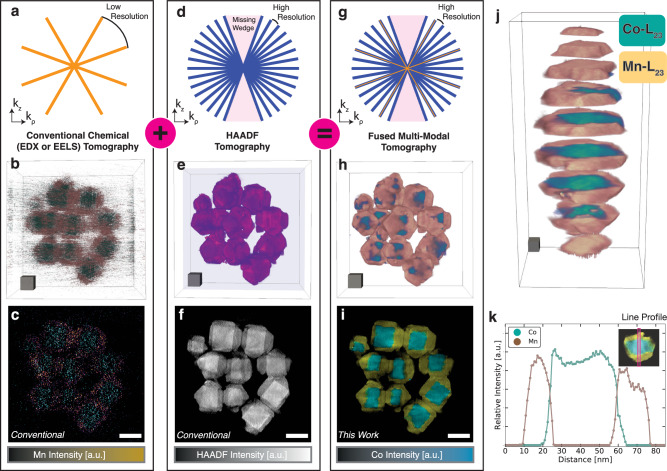
Nanoscale recovery of Co_3_O_4_-Mn_3_O_4_ core-shell nanoparticles. **a**–**c** Raw EELS reconstruction for the Co (blue-green) and Mn (orange) L_2,3_ core-loss edges. **d**–**f** The HAADF tomogram of Co_3_O_4_-Mn_3_O_4_ nanoparticle tracks the structure of the specimen but fails to describe materials chemistry in 3D. **g**–**i** The fused multi-modal reconstruction. Scale cubes, 25 nm^3^. **a**, **d**, **g** Representation in Fourier space of the projections used to reconstruct the tomograms. **j** Fused multi-modal tomogram of a single Co_3_O_4_-Mn_3_O_4_ nanoparticle. Scale cube, 10 nm^3^. **k** A line profile showing the average intensity across the diameter of the particle.

The improvement in reconstruction quality with fused multi-modal chemical tomography (Fig. 2i) is dramatic when compared to traditional chemical tomography (Fig. 2c).

### 3D chemistry at high-resolution, low-dose

In tomography, 3D resolution is described by the Crowther criterion, which states resolution is limited by the object size and the number of specimen projections measured^40^—higher resolution requires more projections^41^. For traditional chemical tomography, few chemical projections are collected and the Crowther relation devastates resolution in 3D. This limitation occurs from the high-dose requirements of chemical mapping (i.e., EDX, EELS) where only a few projections can be collected before radiation damage alters the specimen structure.

Figure 2 shows how specimen projections from each modality are superimposed as planes of information in Fourier space. Chemical tomography is sparsely sampled in Fourier space (Fig. 2a), which results in a tomographic reconstruction containing artifacts and low SNR (Fig. 2b, c). Despite the poor quality, traditional chemical tomography tracks the chemical distribution, and the Mn shell (orange) can be seen surrounding the Co core (blue-green). In contrast, elastically scattered electrons collected by the HAADF detector provide high signals at lower doses and allow many projections to be collected—in practice, HAADF sampling is five to ten times more finely spaced than chemical (Fig. 2d)^32^. The dose required for a single HAADF projection is 10^2^–10^3^ times lower than a chemical projection acquired using core-energy loss spectroscopy. Thus, it is favorable to acquire more HAADF images and achieve higher resolution. Although HAADF tomography permits high-resolution and high-SNR reconstructions of structure, it lacks chemical specificity. This is seen in Fig. 2e, f where the structure is well defined with low noise but the Co and Mn regions are not identifiable.

Exploiting shared information in both modalities, multimodal tomography achieves a chemical resolution in 3D comparable to high-resolution HAADF reconstructions. Although few chemical measurements pose a severely underdetermined problem, fusing with the HAADF modality fills in missing chemical information. This is reflected in Fig. 2g where many HAADF projections (e.g., 50–180) are measured while far fewer chemical projections (e.g., 5–15) are intermittently measured. In this reconstruction, 9 EELS maps and 45 HAADF projections (50–200 mrad detector inner and outer semi-angles) were collected over a ±60 ° tilt range using a 2.4 Å probe with a 24.3 nm depth of focus (300 keV acceleration voltage, 10 mrad convergence angle). High-resolution 3D chemistry is visible in the core-shell Co_3_O_4_-Mn_3_O_4_ using multi-modal tomography in Fig. 2h, i.

Fused multi-modal electron tomography provides unique insight for studying heterostructured nanocrystals with unprecedented geometries. In the case of Co_3_O_4_-Mn_3_O_4_ nanocrystals, the manganese oxide shell is divided into several ordered grains that grow on each surface plane for the cobalt oxide nanocube core^28^. However, the core and shell interface can vary per plane driven by the growth interplay between strain and surface energy, resulting in the formation of grain boundaries^42^. The complete 3D distribution of Co and Mn at the surface and interface is difficult to discern with 2D projected EELS maps or HAADF reconstructions. Fortunately, the fused chemical distributions reveal surface coverage of the shell grains and cross-sections quantify the shell thickness and interface chemistry (Fig. 2k). To further demonstrate, fused multi-modal EELS tomography was used to discern between ZnS and Cu_0.64_S_0.36_ phases (Supplementary Fig. 4) in a heterostructured nanocrystal^29^ and EDX tomography to identify Cu nanoparticles embedded in SiC catalysts (Supplementary Fig. 5).

Data fusion eliminates noticeable noise in the final 3D chemical reconstruction without a loss of resolution. This noise reduction accompanies a dose reduction of roughly 100 fold. Linking the chemical projections to the high SNR HAADF signals dose-efficiently boosts the chemical specificity. Even at modest HAADF signals (e.g., SNR ≃ 10), multi-modal tomography notably outperforms traditional chemical tomography (Supplementary Fig. 21). To illustrate, in Fig. 2, matching the resolution of fused multi-modal chemical tomography using traditional methods would require 45 EELS maps—a fivefold dose increase. However, the SNR of each chemical projection would still fall short (Supplementary Fig. 19) and require roughly 20-times additional dose. In total, multi-modal chemical tomography performs well at one-hundredth the dose requirement of traditional methods.

Reduction of electron beam dose produces irreplaceable advantages for electron tomography—both in terms of accessible resolution and the range of materials classes that can be imaged in 3D. Dose requirements for tomography scale quickly with higher resolution (resolution ∝ dose^−4^)^43,44^. For 3D chemical imaging, multi-modal electron tomography notably improves the sampling and dose constraints that limit resolution across a range of radiation-sensitive materials (See Supplementary Figs. 6, 7).

### Sub-nanometer chemical resolution in 3D

**Fig. 3:**
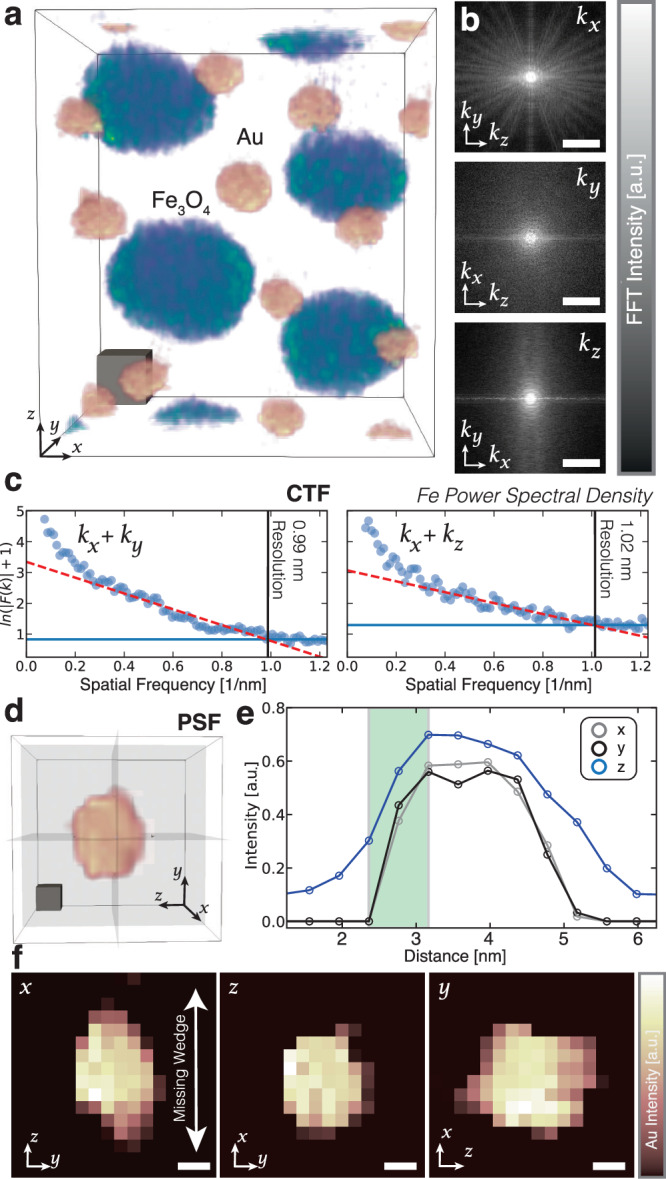
Resolution analysis of Au-Fe_3_O_4_ superlattice nanoparticles. **a** Fused EELS tomograms of Au-Fe_3_O_4_ nanoparticles. Scale cube, 2 nm^3^. **b** Power spectral density of the Fe reconstruction along the principal axial directions shown on the right. Scale bar, 0.5 nm^−1^. **c** Power spectral density profiles for *k*_*x*_-*k*_*y*_ and *k*_*x*_-*k*_*z*_ directions. **d** A 2.5 Au nanoparticle is shown with, **e**, line profiles showing a resolution of 0.8 nm, 0.8 nm, and 1.1 nm along the x, y, and z directions. **f** Planar cross sections of the 2.5 nm. Au nanoparticle.

3D resolution of the chemical distribution in Au-Fe_3_O_4_ nanoparticle superlattice (Fig. 3a) is demonstrated at or below 1 nm using multi-modal tomography. The achieved resolution is quantified in real and reciprocal space. In real space, the resolution limit is verified by visually inspecting a single 3 nm Au nanoparticle (Fig. 3d). The edge sharpness between the reconstructed nanoparticle and vacuum is visibly less than 1 nm. From line profiles, the half-pitch resolution is 0.8 nm × 0.8 nm × 1.1 nm along the *x*, *y*, and *z* directions, respectively. Along optimal directions (*x*, *y*) the resolution is comparable to the Nyquist frequency (8.05 Å). The real-space resolution is consistent with reciprocal space estimates of the cutoff frequency at which the signal drops to the noise floor^1^. Figure 3b highlights power spectral density variations projected on three orthogonal planes. Measured power spectral density along the *k*_*x*_-*k*_*y*_ and *k*_*x*_-*k*_*z*_ directions show information transfer roughly occurring at 0.99 nm and 1.02 nm respectively (Fig. 3c). These directions conservatively represent the 3D resolution from an average of the high-resolution and low-resolution (z-axis) directions. This 3D chemical resolution nearly matches the 3D HAADF resolution 1.00 nm, 1.01 nm in Fig. 3 (Supplementary Fig. 8). For fused multi-modal chemical tomography, the HAADF 3D resolution provides an upper bound to the highest obtainable 3D chemical resolution. A reduction of resolution along the *z*-axis is expected from the incomplete tilt range that creates a missing wedge of information in Fourier space^45^. Avoiding this anisotropic resolution loss has been demonstrated by acquiring a full tilt range (±90°) through the preparation of needle wire samples or preparing nanoparticles on carbon nanofibers^46,47^. Here, we observe approximately a 25% reduction in resolution along the missing wedge direction of the multi-modal chemical reconstruction (Fig. 3e, f).

### Influence of sampling

**Fig. 4:**
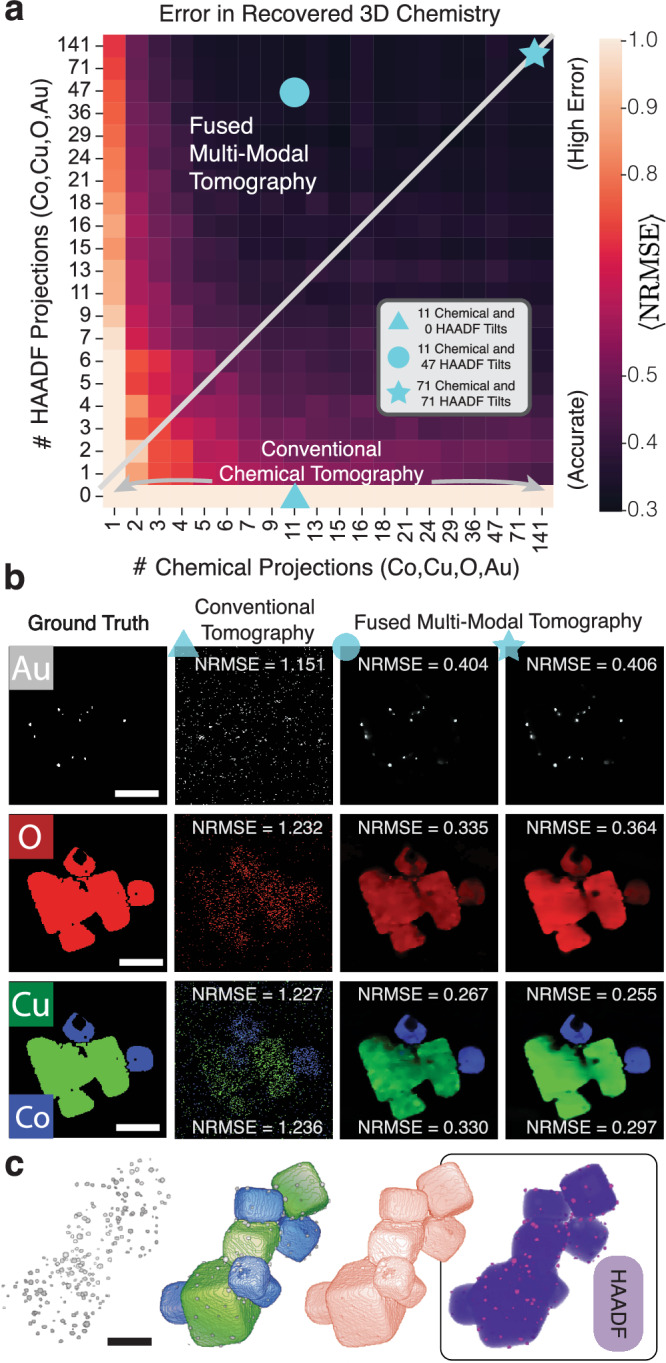
Estimating sampling requirements for accurate recovery with synthetic CoO/CuO nanocubes. **a** An normalized root mean square error (NRMSE) map representing the reconstruction error as a function of the number of HAADF and chemical tilts. Brighter pixels denote results containing incorrect reconstructions from the ground truth. **b** Visualization of three points corresponding to conventional chemical tomography (reconstruction without the HAADF modality), and low or high-dose fused multi-modal electron tomography. **c** The 3D models used for generating synthetic chemical and ADF projections^85^. Scale bar, 75 nm.

Electron tomography simulations show a 3–5 fold improvement in the normalized root mean square error $\left(\langle \,{{\mbox{NRMSE}}}\,\rangle \right)$ averaged across all elements when multi-modal tomography is used over conventional chemical tomography. In Fig. 4 synthetic gold decorated CoO/CuO nanocubes inspired by real experimental data^47^ provide a ground truth comparison to assess the accuracy of fused multi-modal tomography. Simulated projection images are generated from a simple linear incoherent imaging model of the 3D chemical composition with added Poisson noise (See Methods). The specimen tilt range is limited to ±70° to better match typical experimental conditions. The advantages of multi-modal tomography are clearly visible in the 2D slices (Fig. 4b) taken from 3D reconstructions obtained by conventional chemical tomography $\left(\langle \,{{\mbox{NRMSE}}}\,\rangle=1.301\right)$ and fused multi-modal tomography $\left(\langle \,{{\mbox{NRMSE}}}\,\rangle=0.33\right)$. For all chemistries (Au, O, Cu, Co) fused multi-modal tomography is more consistent with the ground truth with higher resolution and reduced noise.

For any number of chemical projections acquired, we see a notable reduction in NRMSE when HAADF projections are integrated into the chemical reconstruction. Figure 4 shows the improved fused multi-modal reconstruction accuracy across a wide range of HAADF and chemical projections for the gold-decorated CoO/CuO nanocubes. The reconstruction error (average NRMSE) across most of the multi-modal parameter space is less than 0.40 compared to values around 1.2 for conventional tomography. Pixel values on the diagram (Fig. 4a) represent the average NRMSE across all elements. This NRMSE map shows data fusion strongly benefits by increasing the HAADF information available. It requires substantially less dose to increase the HAADF projections (i.e., moving vertically on the map) compared to increasing the chemical projections (i.e., moving horizontally on the map).

Conventional chemical tomography does not use HAADF projections (bottom row, Fig. 4a) resulting in an average reconstruction error larger than the entire multi-modal regime. In practice, fused multi-modal tomography is performed in the regime with equal or more HAADF projections than chemical (i.e., top-left triangle). Multi-modal tomography also performs well when the chemical projections exceed the number of HAADF projections, however, this is not practical since HAADF signals can be acquired simultaneously with EDX and EELS. Similar trends are observed in a second large-scale simulation performed on a synthetic composite structure composed of transition metal CoO nanoparticles embedded in a NiO support (Supplementary Fig. 10).

Fused multi-modal electron tomography can measure 3D stoichiometry without knowledge of inelastic cross-sections. The ratio of chemical concentrations for each voxel quantifies local stoichiometry. For the simulated CoO-CuO nanocubes, values agree with the ground truth (Supplementary Fig. 11)—concentrations of Cu, Co, and O are centered at the expected value of 0.50. Here, stoichiometric precision of multi-modal tomography (*σ* = 0.04) is four times better than traditional chemical tomography (*σ* = 0.17). For experimental Fe_3_O_4_ nanoparticles (Supplementary Fig. 12), multi-modal tomography produces an average Fe concentration of 0.46 (0.43 expected) with a standard deviation of 0.15. Note, determining stoichiometry using traditional chemical tomography also requires accurate calculation of the inelastic cross-sections for each experiment^48^.

## Discussion

Although this work presents significant advantages for fused multi-modal electron tomography, the technique requires supervision. Convergence should be verified and reasonable weights for each term in the cost function (Eq. (1)) should be assigned (Supplementary Fig. 13). Error when implementing routine spectroscopic pre-processing (e.g., incorrect background subtraction of EELS spectra^49^ or failure to decouple overlapping characteristic X-ray peaks) will cause inaccurate stoichiometric quantification. These errors will amplify when applied to multi-modal data fusion. Although lighter elements have smaller elastic cross-sections, they tend to have larger inelastic cross-sections which benefits chemical tomography. For example, the K-shell cross-section (chemical spectroscopic signal) of Carbon (*Z* = 6) is over 20-fold larger than Germanium (*Z* = 32)^50,51^. EELS is advantageous for discerning lighter elements whereas overlapping peaks may occur in EDX. In general, electron tomography is favorable for measuring volumes in the range of (10 nm)^3^ to (1000 nm)^3^ at resolutions around 3 to 30 Å^4^. Thick specimens with dimensions that far exceed the mean free path of the electron can produce inversion contrast that will cause electron tomography to fail^52^—also causing failure for multi-modal electron tomography (Supplementary Fig. 14). Electron tomography performs best for thicknesses less than three times the incident electron’s mean free path (e.g., < 550 nm for Silicon at 300 keV)^53^. In all electron tomography experiments, beam convergence angles should be chosen to match the desired resolution and depth of focus^41^. As shown for 2D fused multi-modal electron microscopy^27^, fused multi-modal tomography works best when elements have discernible contributions to the HAADF contrast and all chemical elements have been imaged. Multi-modal tomography leverages CS (e.g., TV min.) which assumes incoherence (i.e., a high level of dissimilarity) between the sensing and sparsifying transform^54–56^—although this assumption typically holds as demonstrated for the datasets presented herein.

In summary, we present fused multi-modal electron tomography that enables chemically-sensitive 3D reconstruction of matter with nanometer resolution at high SNR. We demonstrate that researchers do not need to choose between measuring 3D structure without chemical detail or characterizing chemistry along a single viewing direction. By linking signals from elastic (HAADF) and inelastic (EDX/EELS) scattering processes, the traditional dose limits of chemical tomography are substantially surpassed. In some cases, a one-hundred-fold reduction in dose is estimated. When compared to 2D chemical imaging^27^, we show the benefits of data fusion in 3D are much greater. To demonstrate, the complete volumetric density of each chemistry was mapped in several systems including Au-Fe_3_O_4_, Co_3_O_4_-Mn_3_O_4_, ZnS-Cu_0.64_S_0.36_, and Cu-SiC nanomaterials. In both artificial and experimental datasets, fused multi-modal electron tomography shows significant benefits in the accuracy of 3D chemical imaging. This approach enables chemical tomography of a wide range of previously inaccessible materials with moderate radiation sensitivity. At chemical resolutions of 1 nm, fused multi-modal electron tomography will facilitate understanding of geometrically complex materials—from 3D semiconductor gate stacks^57^, clean energy materials^58,59^, or photoluminescence quantum dot nanoparticles^60^.

Here, fused multi-modal tomography used commonly available STEM detectors (HAADF, EDX, and EELS), however, future approaches can further integrate other modalities—such as 4D-STEM from pixel-array detectors^61^, annular bright field^62^, ptychography^63^, low-loss EELS^64^. Furthermore, the tremendous potential of multi-modal data fusion as a paradigm readily enhances deep learning to capitalize on the unique advantages from both domains^65^.

## Methods

### Specimen synthesis and preparation

#### Au-Fe_3_O_4_ superlattice nanoparticles

Syntheses of 3.9 nm Au NPs^66^ and 10.2 nm Fe_3_O_4_ NPs^67^ were carried out under nitrogen atmosphere using standard Schlenk line techniques according to literature methods. Polystyrene-based ligands were attached to the NP surface through a ligand exchange process as reported before^68^. Thiol-terminated PS (PS-SH) was used as the polymeric ligand for Au NPs and was synthesized using Radical Addition Fragmentation Transfer polymerization and end-functionalized by aminolysis. Amine-terminated polystyrene was used as the polymeric ligand for Fe_3_O_4_ NPs and was synthesized using atom transfer radical polymerization and end-group modification^69^. Binary superlattice of Au and Fe_3_O_4_ NPs was prepared by nanoparticle co-crystallization at water-air interface. A toluene solution containing two types of NPs with concentration ratio of 2:1 was drop-cast onto the water surface in a Teflon well and slowly dried overnight. The binary nanoparticle film was transferred onto a 200-mesh carbon TEM substrate and further dried in vacuum oven for 6 h to remove residual solvent.

#### Co_3_O_4_ nanocubes

A mixture of 0.37 g of cobalt(II) perchlorate (Aldrich) and 2.7 g of oleylamine (Acros) in 15 mL of 1-octanol (Aldrich) was heated to 120 °C under air and aged for 2 h. During the heating, 0.7 mL of distilled water was added before the temperature reaches 120 °C. After the reaction, an excess amount of acetone and ethanol was added and Co_3_O_4_ nanocubes were retrieved by centrifugation.

#### Core-shell Co_3_O_4_-Mn_3_O_4_ nanoparticles

An organic/aqueous suspension was prepared by adding 0.080 g of Co_3_O_4_ nanocubes into a mixture of oleylamine (5 mmol), oleic acid (0.5 mmol), formic acid (3.15 mmol, Aldrich), and 15 mL of xylenes (Aldrich). The as-prepared suspension was heated to 40 °C under air and aged for 3 h with magnetic stirring. And then, 0.7 mL of 0.7 M aqueous solution of manganese (II) chloride tetrahydrate was rapidly injected into the suspension at 90 °C and aged for 1.5 h under air. After the reaction, the nanocrystals were washed with hexane/ethanol and retrieved by centrifugation. The final product was prepared with three iterations of this process.

#### ZnS-Cu_0.64_S_0.36_ nanocrystals

Synthesis of the ZnS-Cu_0.64_S_0.36_ Heterostructured NPs was performed as described by literature using typical air and water free synthetic techniques^29^. Cu_1.81_S (roxbyite) nanocrystals are synthesized by first dissolving CuCl_2_⋅2H_2_O in oleylamine (OLAM) at 200 °C after thoroughly degassing the solution at high temperature. Tert-butyl-disulfide is then injected at 180 °C and the reaction continues at this temperature for 40 min. After cooling to room temperature, the NPs are washed with hexanes and acetone then dried in a vacuum desiccator. The roxbyite NPs are then injected into a concentrated Zn ion solution heated at 50 °C for 10 min to facilitate the reaction. Briefly, ZnCl^2^ and OLAM are degassed at high temperature and then heated at 180 °C for 30 min to make a concentrated solution of Zn^2^+ for cation exchange. After cooling the Zn^2+^ solution to 100 °C, an aliquot of the solution is mixed with toluene and the temperature is adjusted to 50 °C. The synthesized roxbyite NPs are dissolved in tri-octyl phosphine and then injected into the Zn^2+^ solution in and allowed to react for 30 min before quenching the reaction with cold acetone.

#### Cu-SiC catalyst

The Cu/SiC catalyst was prepared on a commercial SiC support purchased from a commercial vendor with a Brunauer-Emmett-Teller surface area of 30 m^2^/g and pore volume of 0.4 cm^3^/g. following previously described methods^70^. The catalyst was prepared by incipient wetness impregnation using a 2M Cu(NO_3_)_2_⋅3H_2_O aqueous solution followed by drying (120 °C for 2 h) and calcination in air (350 °C for 2 h) at a heating rate of 2 deg/min.

#### Acrylic C-TiO_2_ nanoparticles

The C-TiO_2_ sample was prepared by blending commercial TiO_2_ particles (purchased from Chemours) with an emulsion polymer latex. Before conducting the chemical imaging at room temperature, the blend was pre-treated under the electron beam in a Thermo Fisher T12 TEM at −80 °C to promote cross-linking in the latex and preserve its morphology above the glass transition temperature.

### Electron tomography acquisition

Simultaneously acquired HAADF and EELS tilts series for the Au-Fe_3_O_4_ specimen were collected on a Talos F200X G2 (Thermo Fisher) operated at 200 keV with a probe current of 115 pA, probe semi-angle of roughly 10.5 mrad and inner collection semi-angle of 50 mrad. The HAADF projections were collected from −60° to +60° with a 3° angular increment using a Model 2021 Fischione Analytical Tomography Holder. At each tilt angle, a STEM image with a 24 μs dwell time at each pixel of a lateral dimension 6.4 Å. Simultaneously acquired HAADF and EELS spectrums were acquired at acquired with a 15 ° angular increment with a dwell time of 3 ms receiving a total electron dose of 4.9 × 10^5 ^e/Å^2^ (1.72 × 10^4 ^e/Å^2^, 4.73 × 10^5 ^e/Å^2^ for the HAADF and EELS modality, respectively). Refer to Supplementary Figs. 15, 16 to view the raw tilt series.

Simultaneously acquired HAADF and EELS tilt series for the Co_3_O_4_-Mn_3_O_4_ specimen were collected on a double aberration-corrected modified FEI Titan 80–300 microscope (the TEAM I instrument at the National Center for Electron Microscopy within Lawrence Berkeley National Laboratory) operated at 300 keV with a probe current of 115 pA and semi-angle of roughly 10 mrad. This microscope is equipped with a Gatan K3 detector and Continuum spectrometer. The HAADF projections were recorded from −60° to +60° with a 3° angular increment using a Hummingbird Scientific eucentric Tomography Holder. At each tilt angle, a STEM image with a 24 μs dwell time at each pixel of a lateral dimension of 7.79 Å. Simultaneously acquired HAADF and EELS spectrums were acquired at acquired with a 15 ° angular increment with a dwell time of 0.677 ms receiving a total electron dose of 8.37 × 10^4 ^e/Å^2^ (1.16 × 10^4 ^e/Å^2^, 7.21 × 10^4 ^e/Å^2^ for the HAADF and EELS modality, respectively). Refer to Supplementary Figs. 17, 19 to view the raw tilt series.

Simultaneously acquired HAADF and EDX tilt series for the Cu-SiC specimen were collected on a Talos F200X G2 (Thermo Fisher) operated at 200 keV with a probe current of 250 pA, probe semi-angle of roughly 10.5 mrad and collection angle of 44–200 mrad. The HAADF projections were collected from −75 to +70 with a 3° angular increment. At each tilt angle, a STEM image with a 20 μs at each pixel of the lateral dimension of 1.4679 nm. Simultaneously acquired HAADF and EDX spectrums were acquired at acquired with a 15 ° angular increment with a dwell time of 20 μs dwell time for 25 frames receiving a total electron dose of 4.33 × 10^4 ^e/Å^2^ (7.1 × 10^3 ^e/Å^2^, 3.62 × 10^4 ^e/Å^2^ for the HAADF and EELS modality, respectively). The initial chemical distributions were generated from EDX maps using commercial Velox software that produced initial net count estimates (however atomic percent estimates are also suitable).

### Multi-modal tilt series alignment

The EELS signals were obtained by integration over the core loss edges, all of which were done after background subtraction. The background EELS spectra were modeled using a linear combination of power laws implemented using the open-source Cornell Spectrum Imager software^9^.

Before tilt series alignment, the spectrum images have been drift-corrected after acquisition assuming a time-dependent linear drift model, as illustrated in Supplementary Fig. 20. The survey image, which is taken with an identical dwell time as the HAADF tilts, is taken as a reference. Iterative image registration between the chemical and HAADF signals seek an optimal translation and affine transformation. Following registration, the background of each projection was removed. For this purpose, the mean gray level in the outer regions was calculated for each projection and subtracted. In this way, the signal contribution of the carbon film could be eliminated.

For the alignment of the tilt series, a coarse alignment is performed with either the center of mass (CoM) or cross-correlation method^71^. CoM works best when the total projected volume is fixed across specimen tilts (i.e., the object is isolated)^72^. In cases where either of these requirements are not met (e.g., fields of view where multiple particles are visible as demonstrated with the Au-Fe_3_O_4_ nanoparticles), cross-correlation should be considered. Fine alignment is performed with custom written projection matching method^73^ on the HAADF modality. The measured translation shifts are subsequently applied to the corresponding tilts where simultaneously acquired chemical maps were acquired.

### Fused multi-modal tomography recovery

Here, fused multi-modal electron microscopy is framed as an inverse problem expressed in the following form: $\hat{{{{{{{{\boldsymbol{x}}}}}}}}}\,=\,\arg \mathop{\min }\nolimits_{{{{{{{{\boldsymbol{x}}}}}}}}\ge 0}{\lambda }_{1}{\Psi }_{1}({{{{{{{\boldsymbol{x}}}}}}}})+{\lambda }_{2}{\Psi }_{2}({{{{{{{\boldsymbol{x}}}}}}}})+{\lambda }_{3}{{{{{{{\rm{TV}}}}}}}}({{{{{{{\boldsymbol{x}}}}}}}})$ where $\hat{{{{{{{{\boldsymbol{x}}}}}}}}}$ is the final reconstruction, and the three terms are described in the main manuscript (Eq. (1)). When implementing an algorithm to solve this problem, we concatenate the multi-element spectral variable (***x***) as 2D matrices: ${{{{{{{\boldsymbol{x}}}}}}}}\in \,{{\mathbb{R}}}^{{n}_{y}\cdot {n}_{y}\cdot {n}_{i}\times {n}_{x}}$ where *n*_*i*_ denotes the total number of reconstructed elements and *n*_*x*_, *n*_*y*_ represent number of pixels in the x and y direction and ***x***_*i*_, ***b***_*i*_ are the reconstructions and chemical maps for element *i* (${{{{{{{{\boldsymbol{x}}}}}}}}}_{i}\in \,{{\mathbb{R}}}^{{n}_{y}\cdot {n}_{y}\times {n}_{x}}$ and ${{{{{{{{\boldsymbol{b}}}}}}}}}_{i}\in \,{{\mathbb{R}}}^{{n}_{y}\cdot {N}_{{{{{{{{\rm{chem}}}}}}}}}^{{{{{{{{\rm{proj}}}}}}}}}\times {n}_{x}}$). Here the axis of rotation is along the *x*-direction (*n*_*x*_).

The optimization problem is solved by a combination of gradient descent with TV regularization. We minimize this cost function by iteratively descending along the negative gradient directions for the first two terms and subsequently evaluate the isotropic TV proximal operator to denoise the chemical volumes^74^. The gradients of the first two terms are:

2 $${\nabla }_{{{{{{{{\boldsymbol{x}}}}}}}}}{\Psi }_{1}({{{{{{{\boldsymbol{x}}}}}}}})=-\gamma \,{{\mbox{diag}}}\,\left({{{{{{{{\boldsymbol{x}}}}}}}}}^{\gamma -1}\right){{{{{{{{\mathbf{\Sigma }}}}}}}}}^{T}{{{{{{{{\boldsymbol{A}}}}}}}}}_{h}^{T}\left({{{{{{{{\boldsymbol{A}}}}}}}}}_{h}{({{{{{{{\mathbf{\Sigma }}}}}}}}{{{{{{{{\boldsymbol{x}}}}}}}}}^{\gamma })}^{T}-{{{{{{{{\boldsymbol{b}}}}}}}}}_{H}\right)$$

3 $${\nabla }_{{{{{{{{{\boldsymbol{x}}}}}}}}}_{i}}{\Psi }_{2}({{{{{{{{\boldsymbol{x}}}}}}}}}_{i})={{{{{{{{\boldsymbol{A}}}}}}}}}_{c}^{T}\left(({{{{{{{{\boldsymbol{A}}}}}}}}}_{c}{{{{{{{{\boldsymbol{x}}}}}}}}}_{i}-{{{{{{{{\boldsymbol{b}}}}}}}}}_{i})\oslash ({{{{{{{{\boldsymbol{A}}}}}}}}}_{c}{{{{{{{{\boldsymbol{x}}}}}}}}}_{i}+\varepsilon )\right),$$

where ⊘ denotes point-wise division, ${{{{{{{{\boldsymbol{b}}}}}}}}}_{H}\in {{\mathbb{R}}}^{{n}_{y}{N}_{{{{{{{{\rm{HAADF}}}}}}}}}^{{{{{{{{\rm{proj}}}}}}}}}\times {n}_{x}}$ are the HAADF measurements, ${{{{{{{{\boldsymbol{A}}}}}}}}}_{h}\in {{\mathbb{R}}}^{{n}_{y}\cdot {N}_{{{{{{{{\rm{HAADF}}}}}}}}}^{{{{{{{{\rm{proj}}}}}}}}}\times {n}_{y}\cdot {n}_{y}}$ and ${{{{{{{{\boldsymbol{A}}}}}}}}}_{c}\,\in \,{{\mathbb{R}}}^{{n}_{y}\cdot {N}_{{{{{{{{\rm{chem}}}}}}}}}^{{{{{{{{\rm{proj}}}}}}}}}\times {n}_{y}\cdot {n}_{y}}$ are forward projection matrices operating on the chemical and HAADF modalities. Here, the first term in the cost function, relating the elastic and inelastic modalities, has been equivalently re-written as ${\Psi }_{1}=\frac{1}{2}\parallel {{{{{{{{\boldsymbol{A}}}}}}}}}_{h}({{{{{{{\mathbf{\Sigma }}}}}}}}{{{{{{{{\boldsymbol{x}}}}}}}}}^{\gamma })-{{{{{{{{\boldsymbol{b}}}}}}}}}_{H}{\parallel }_{2}^{2}$, where ${{{{{{{\mathbf{\Sigma }}}}}}}}\in {{\mathbb{R}}}^{{n}_{y}\cdot {n}_{y}\times {n}_{y}\cdot {n}_{y}\cdot {n}_{i}}$ and **Σ** ***x*** expresses the summation of all chemistries as matrix-vector multiplication. Evaluating the TV proximal operator is in itself another iterative algorithm. In addition, we impose a non-negativity constraint since negative concentrations are unrealistic. We initialize the first iterate with reconstructions composed purely of the raw measured data (${{{{{{{{\boldsymbol{x}}}}}}}}}_{i}^{0}=\arg \min {\Psi }_{2}$). This is an ideal starting point as it is a local minimizer of Ψ_2_.

Appropriate step sizes for convergence of Eq. (1) can be determined estimating the Lipschitz constant of the measurement matrix using the Power method^75^. Convergence can be confirmed by assessing each term in the cost function as the reconstruction proceeds (Supplementary Fig. 13). Sub-optimal parameters often result in slower convergence. Smooth and asymptotic decay of all three terms in Eq. (1) is an indicator of reliable reconstruction. We find that the optimal weights (*λ*) in Eq. (1) do not change significantly between datasets and even sub-optimal terms outperform traditional tomography methods. However careful selection can also be achieved by selecting values within the inflection point of the Pareto front in Eq. (1) (see Supplementary Fig. 13)^76^. The final 3D HAADF and multi-modal chemical volumes were rendered using the Tomviz platform (tomviz.org^77^).

### Multi-modal simulations and Bayesian hyperparameter optimization

To demonstrate the functionality of our fused multi-modal electron tomography algorithm, we created a multi-channel phantom specimen inspired from an experimental system. The phantom consists of four channels, which we attribute to the crystal stoichiometry of CuO, CoO, and Au (Fig. 4c) with a volume size of 256^3^. The HAADF intensity is proportional to ${\sum }_{e}{({Z}_{i}{x}_{i})}^{\gamma }$ where *x*_*i*_ reflects the element’s stoichiometry. To produce chemical maps with realistic noise characteristics, we set the background (vacuum) to roughly 15% of the max intensity and subsequently applied Poisson noise to meet the desired SNR. For a Poisson-limited signal, each synthetic image has an SNR of $\frac{{\mu }_{s}+{\mu }_{s}^{2}}{{\sigma }_{N}^{2}}$ where *μ*_*s*_ is the mean signal and ${\sigma }_{N}^{2}$ is the variance of noise^41^ In the case of Fig. 4, the SNR of the Co, Cu, O, Au, and HAADF modalities were 1.92, 2.89, 2.69, 1.96, 2208.67, respectively. Prior to measuring the NRMSE of the reconstructed volumes, the chemical distributions were normalized with zero mean and unit standard deviation. The NRMSE expresses a normalized measure of agreement between the reconstructed (***x***) and ground truth (***y***) : $\sqrt{\frac{{\sum }_{i,j,k}{({{{{{{{{\boldsymbol{y}}}}}}}}}_{i,j,k}-{{{{{{{{\boldsymbol{x}}}}}}}}}_{i,j,k})}^{2}}{{\sum }_{i,j,k}{({{{{{{{{\boldsymbol{y}}}}}}}}}_{i,j,k})}^{2}}}$. While the HAADF SNR may be high, we found the NRMSE reliably converges when above 50 (Supplementary Fig. 21).

Determining optimal regularization parameters for the phase diagram (Fig. 4a) is computationally expensive to explore due to its variability across sampling conditions. While grid search could find the best parameters by exhaustively exploring all possible candidate values, the computation time would be expensive as each map would take ~125 days to complete on a single GPU.

We efficiently explored the parameter space with Bayesian optimization (BO)—a machine learning framework known for optimizing expensive unknown objective functions with minimal evaluations^78,79^. It works by building a probabilistic model of the objective function with Gaussian processes (GP) regression. GP not only estimates our function of interest but also provides the uncertainty measurements to guide future predictions. BO takes into account past evaluations when determining future hyperparameter selections via an acquisition function^80^. For our simulations, we carried out BO with GP in Python with the Scikit Optimize library (scikit-optimize.github.io/stable) with the Matern kernel and GP Hedge acquisition strategy^81^. By exploiting BO with GP, we are able to provide an atlas of balanced hyperparameters for Eq. (1) with the CoNiO and CoCuO synthetic datasets (Supplementary Figs. 22, 23). The estimated parameter landscape is smooth and continuous with a clear global optimum.

Asynchronous parallel BO on supercomputing resources allowed us to efficiently run several reconstructions simultaneously on a single node. This form of parallel computing resulted in several factors of computational speed up as multiple GPUs received unique experimental parameters (e.g., SNR or sampling) to reconstruct concurrently amongst each other. Specifically, the computation time to generate an NRMSE map was reduced by 99.8%—taking less than a day to complete (18 h). In total, 3452 GPU hours were used to complete these simulations—1078 h on Summit - OLCF and 1078 h on ThetaGPU—ALCF for the phase diagrams (Fig. 4 and Supplementary Fig. 10). An additional 1296 GPU hours on Summit were used to produce the SNR plots (Supplementary Fig. 21).

### Supplementary information

Supplementary Information

Peer Review File

## Data Availability

The raw and aligned Au-Fe_3_O_4_, Co_3_O_4_-Mn_3_O_4_, and Cu-SiC tilt series with reconstructed 3D chemistries are available in a Zenodo repository^82^.

## Code availability

All of the multi-modal electron tomography reconstruction and iterative alignment codes are available at github.com/jtschwar/tomo_TV and github.com/jtschwar/projection_refinement^83^. A sample jupyter notebook outlining the fused multi-modal reconstruction on the Cu-SiC and Au-Fe_3_O_4_ material systems will be available in the tomo TV repository.
