# Supplementary material for: Imaging 3D chemistry at 1 nm resolution with fused multi-modal electron tomography
Source: Nat Commun. 2024 Apr 26;15:3555. doi: 10.1038/s41467-024-47558-0 (PMC11053043; doi:10.1038/s41467-024-47558-0)
Supplement: Supplementary file 1 — Supplementary Information [file 41467_2024_47558_MOESM1_ESM.pdf]

Supplementary Information for

Imaging 3D Chemistry at 1 nm Resolution with Fused Multi-Modal Electron Tomography

Jonathan Schwartz, Zichao Wendy Di, Yi Jiang, Jason Manassa, Jacob Pietryga, Yiwen Qian, Min Gee Cho, Jonathan L. Rowell, Huihuo Zheng, Richard D. Robinson, Junsi Gu, Alexey Kirilin, Steve Rozeveld, Peter Ercius, Jeffrey A. Fessler, Ting Xu, Mary Scott, and Robert Hovden

Contents

1 Multi-Modal Reconstruction of Au-Fe_3O_4 Nanoparticles Inside a Carbon Support 3

2 Consequence of Reconstructing CuO/CoO Simulation with Individual Components in Cost Function 4

3 Consequence of Reconstructing CoNiO Simulation with Individual Components in Cost Function 5

4 Multi-Modal EELS Tomography of ZnS - $Cu_{0.64}S_{0.36}$ Heterostructured Nanoparticles 6

5 Multi-Modal EDX Tomography of Cu-SiC Nanoparticles 7

6 Historical Demonstrations of Multi-Element Chemical Tomography 8

7 Resolution and Electron Dose for Multi-Modal Electron Tomography 9

8 Measuring the Resolution of Au-Fe_3O_4 Nanoparticles in the HAADF 10

9 Sampling Requirements of Multi-Modal Tomography on CoO/CuO Nanocubes 11

10 Estimating the Sampling Requirements for a CoNiO Composite 12

11 Measuring 3D Stoichiometric Concentration for simulated CuO-CoO Nanocubes 13

12 Measuring 3D Stoichiometric Concentration of Au-Fe_3O_4 Superlattice Nanoparticles 14

13 Assessing Convergence and Selecting Hyperparameters with Paraeto Front Curves 15

14 Consequence of Reconstructing Thick 280 nm Nanoparticles with EELS Tomography 16

15 Raw HAADF Tilt Series for the Au-Fe_3O_4 Nanoparticles 17

16 Raw EELS Tilt Series for the Au-Fe_3O_4 Nanoparticles 18

17 Raw HAADF Tilt Series for the Co_3O_4 - Mn_3O_4 Core-Shell Nanoparticles 19

18 Raw EELS Tilt Series for the Co_3O_4 - Mn_3O_4 Core-Shell Nanoparticles 20

19 Orthogonal Views of Au - Fe_3O_4 and Co_3O_4 - Mn_3O_4 Nanoparticles 21

20 Drift Correction of Spectrum Maps 22

21 SNR Dependency for Successful Fused Multi-Modal Recovery 23

22 Hyperparameter Estimation with Bayesian Optimization for the CuO-CoO Nanocubes 24

23 Hyperparameter Estimation with Bayesian Optimization for CoNiO Composite 25

24 Fusion Multi-Modal Electron Tomography Pseudo Code 26

1 Multi-Modal Reconstruction of Au-Fe_3O_4 Nanoparticles Inside a Carbon Support

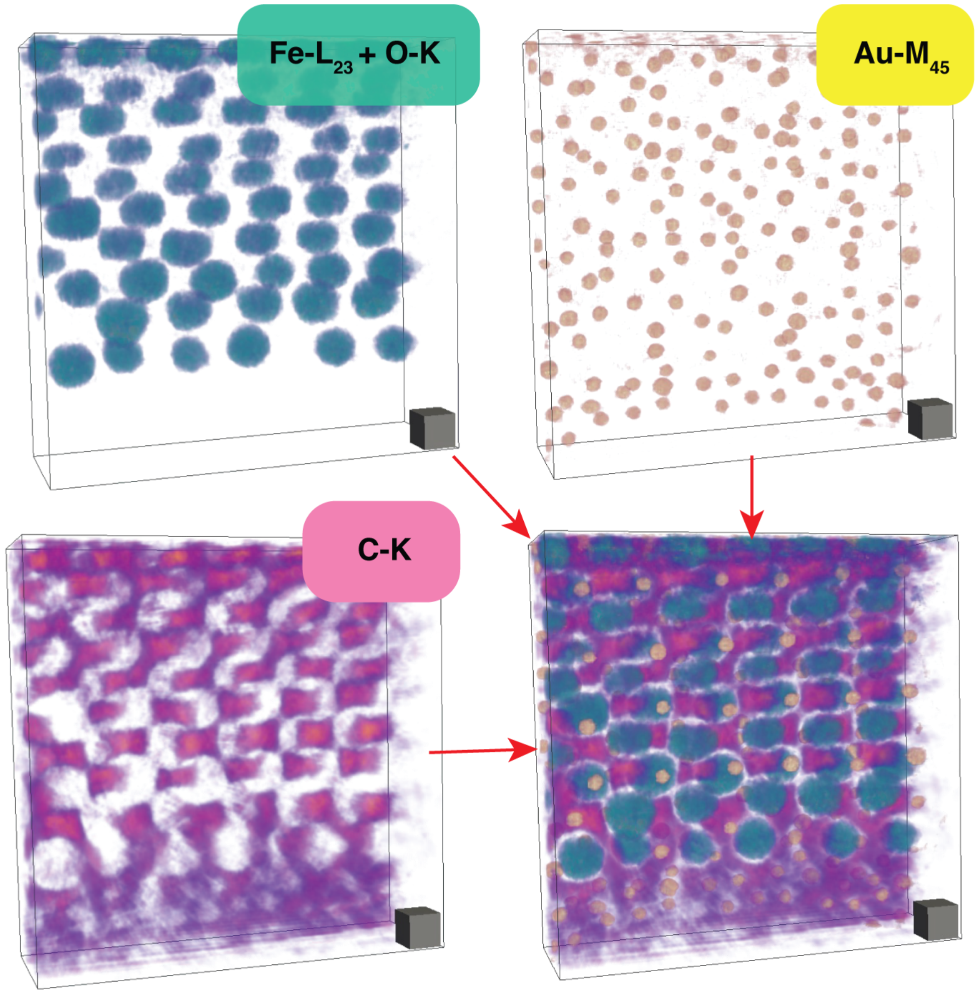

Fig.1 | The complete reconstruction of the Au-Fe_3O_4 nanoparticle superlattice inside the carbon matrix (highlighted in purple). Scale cube, 10 nm^3.

2 Consequence of Reconstructing CuO/CoO Simulation with Individual Components in Cost Function

$$\underset{\boldsymbol{x}_i \geq 0}{\arg\min} \; \overbrace{\frac{\lambda_1}{2}\left\| \boldsymbol{A}_h \sum_i (Z_i \boldsymbol{x}_i)^\gamma - \boldsymbol{b}_H \right\|_2^2}^{\Psi_1(\boldsymbol{x})} + \overbrace{\lambda_2 \sum_i \left(\boldsymbol{1}^T \boldsymbol{A}_c \boldsymbol{x}_i - \boldsymbol{b}_i^T \log(\boldsymbol{A}_c \boldsymbol{x}_i + \varepsilon) \right)}^{\Psi_2(\boldsymbol{x})} + \lambda_3 \sum_i \|\boldsymbol{x}_i\|_{\mathrm{TV}}$$

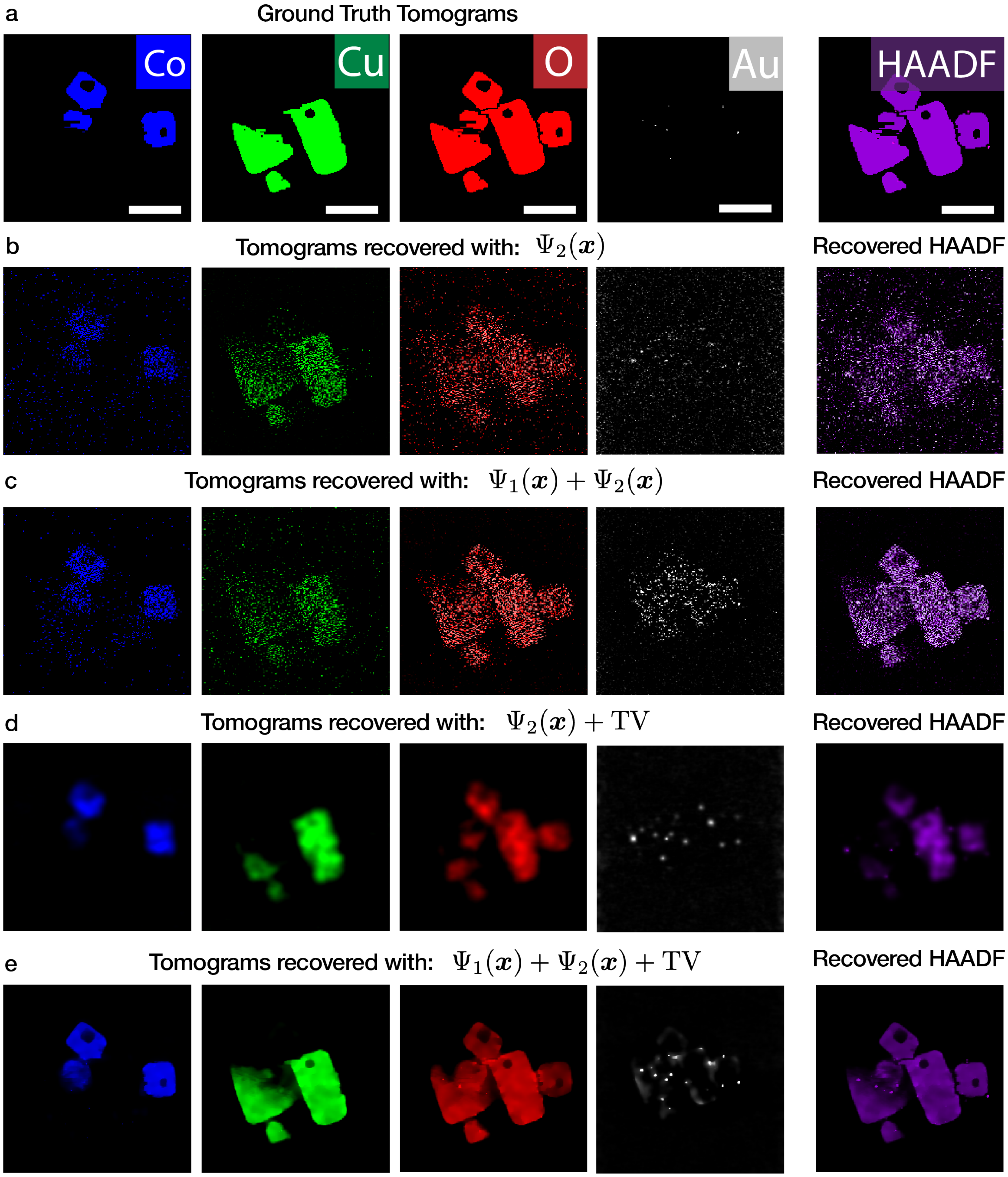

Fig.2 | a Synthetic CuO/CoO nanocubes ground truth EELS maps and simultaneous HAADF reconstruction. **b** Slice of the reconstructed nanocubes dataset with only Ψ_2 (data consistency), λ_1,λ_3=0. The recovered EELS maps and HAADF reconstruction are noisy. **c** Slice of the reconstructed multi-modal nanocubes dataset with Ψ_1 and Ψ_2 (model + data-consistency), λ_3=0. The EELS maps are still noisy but show improvement with the extra Ψ_1 term. **d** Slice of the reconstructed multi-modal nanocubes dataset with Ψ_2 + TV, λ_1=0 . The expression Ψ_2 + TV is equivalent to a denoising problem; thus the resulting reconstructions produce common staircase artifacts associated with TV [1]. **e** Slice of the reconstructed nanocubes dataset with fused multi-modal electron tomography. Scale bar, 75 nm.

3 Consequence of Reconstructing CoNiO Simulation with Individual Components in Cost Function

$$\underset{\boldsymbol{x}_i \geq 0}{\arg\min} \; \overbrace{\frac{\lambda_1}{2} \left\| \boldsymbol{A}_h \sum_i (Z_i \boldsymbol{x}_i)^\gamma - \boldsymbol{b}_H \right\|_2^2}^{\Psi_1(\boldsymbol{x})} + \overbrace{\lambda_2 \sum_i \left(\boldsymbol{1}^T \boldsymbol{A}_c \boldsymbol{x}_i - \boldsymbol{b}_i^T \log(\boldsymbol{A}_c \boldsymbol{x}_i + \varepsilon) \right)}^{\Psi_2(\boldsymbol{x})} + \lambda_3 \sum_i \|\boldsymbol{x}_i\|_{\mathrm{TV}}$$

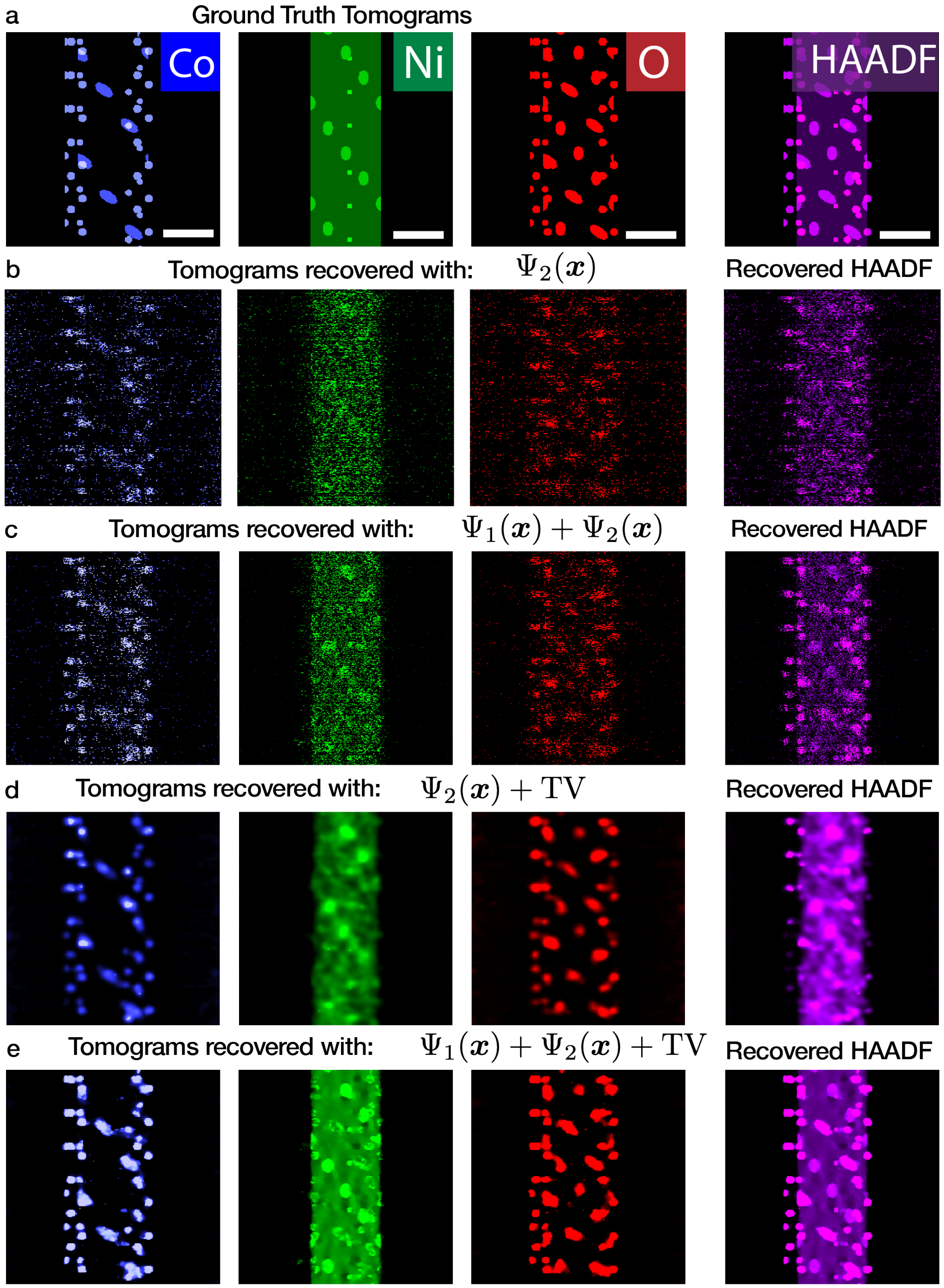

Fig.3 | a Synthetic CoNiO nanotube ground truth EELS maps and simultaneous HAADF reconstruction. **b** Slice of the reconstructed nanotube dataset with only Ψ_2 (data consistency), λ_1,λ_3=0. The recovered EELS maps and HAADF reconstruction are noisy. **c** Slice of the reconstructed multi-modal nanotube dataset with Ψ_1 and Ψ_2 (model + data-consistency), λ_3=0. The EELS maps are still noisy but show improvement with the extra Ψ_1 term. **d** Slice of the reconstructed multi-modal nanotube dataset with Ψ_2 + TV, λ_1=0 . The expression Ψ_2 + TV is equivalent to a denoising problem; thus the resulting reconstructions produce common staircase artifacts associated with TV [1]. **e** Slice of the reconstructed nanotube dataset with fused multi-modal electron tomography. Scale bar, 50 nm.

4 Multi-Modal EELS Tomography of ZnS - $Cu_{0.64}S_{0.36}$ Heterostructured Nanoparticles

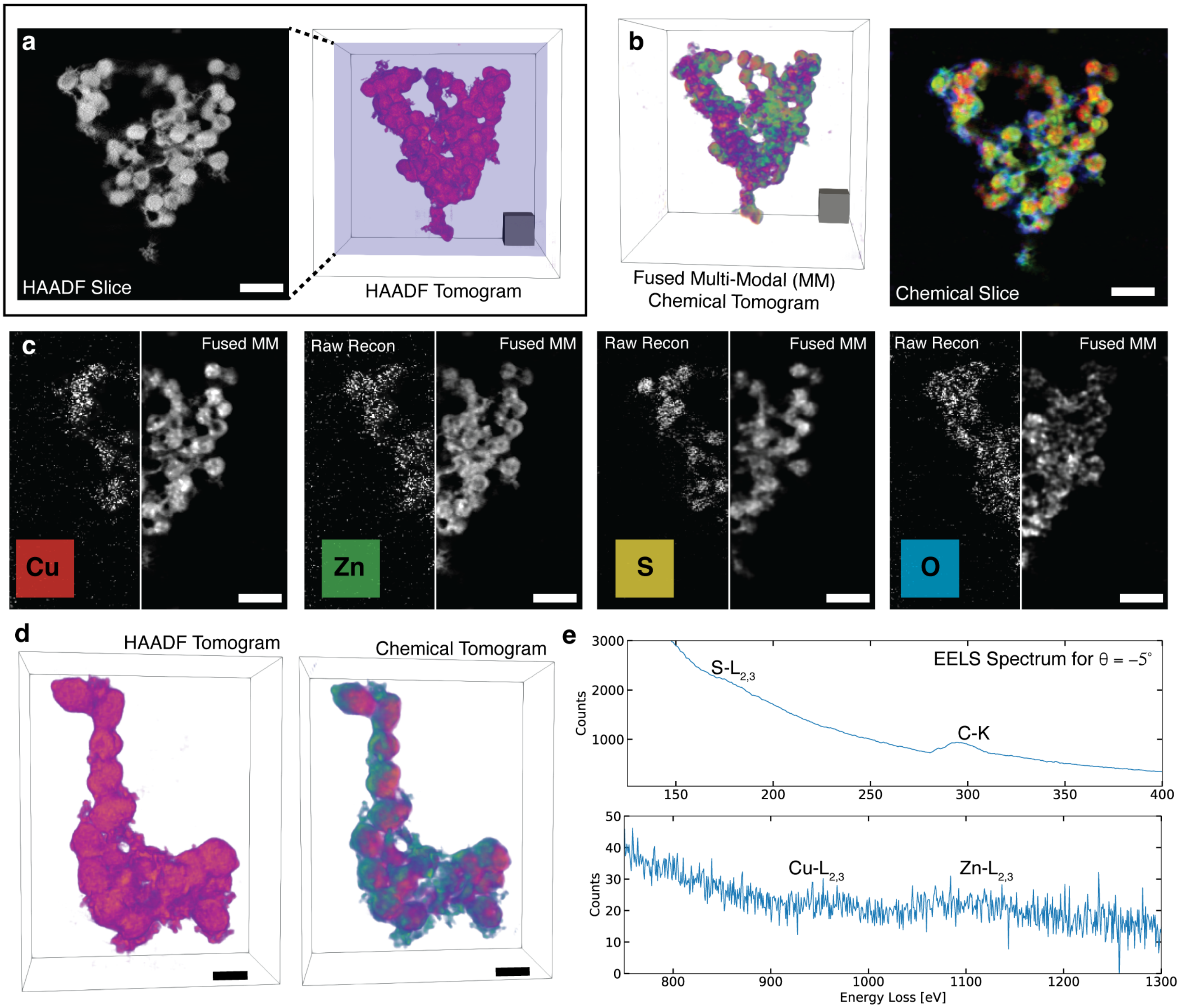

Fig.4 | a HAADF tomography on heterostructured nanocrystals with applications in photovoltaic devices and battery electrodes [2]. The copper sulfide properties are sensitive to stoichiometry and crystal structure at the interface. The HAADF reconstruction and 2D slice are shown on the left. **b** The fused multi-modal reconstruction illustrating CuS or ZnS-rich nanoparticles and oxidized shells. **c** 2D slices of the chemical reconstructions with the noisy traditional reconstructions highlighted on the left of each image. Scale bar, 50 nm. **d** The HAADF and fused multi-modal chemical tomogram for a smaller field of view. **e** Representative EELS spectra for the S, C, Cu, and Zn core loss edges.

The simultaneously acquired HAADF and EELS tilt series for the specimen were collected on a Talos F200X G2 (Thermo Fisher) operated at 200 keV with a probe semi-angle of roughly 10.5 mrad and inner collection semi-angle of 50 mrad. For the larger cluster (**a**-**c**), the HAADF projections were collected from -74° to +70° with a 3° angular increment using a Model 2021 Fischione Analytical Tomography Holder. At each tilt angle, a STEM image with a μs dwell time at each pixel of a lateral dimension of 1.26 nm. Simultaneously acquired HAADF and EELS spectrums were acquired at acquired with a 15° angular increment with a dwell time of 2 ms receiving a total electron dose of 8.25×10^4 e/Å^2 (3.87×10^3, 7.86×10^4 for the HAADF and EELS modality, respectively).

For the smaller cluster (**d**), the HAADF projections were collected from -74° to +67° with a 3° angular increment using a Model 2021 Fischione Analytical Tomography Holder. At each tilt angle, a STEM image with a 30 μs dwell time at each pixel of a lateral dimension of 1.12 nm. Simultaneously acquired HAADF and EELS spectrums were acquired at acquired with a 15° angular increment with a dwell time of 3.5 ms receiving a total electron dose of 2.07×10^5 e/Å^2 (6.87×10^3, 2.01×10^5 for the HAADF and EELS modality, respectively).

5 Multi-Modal EDX Tomography of Cu-SiC Nanoparticles

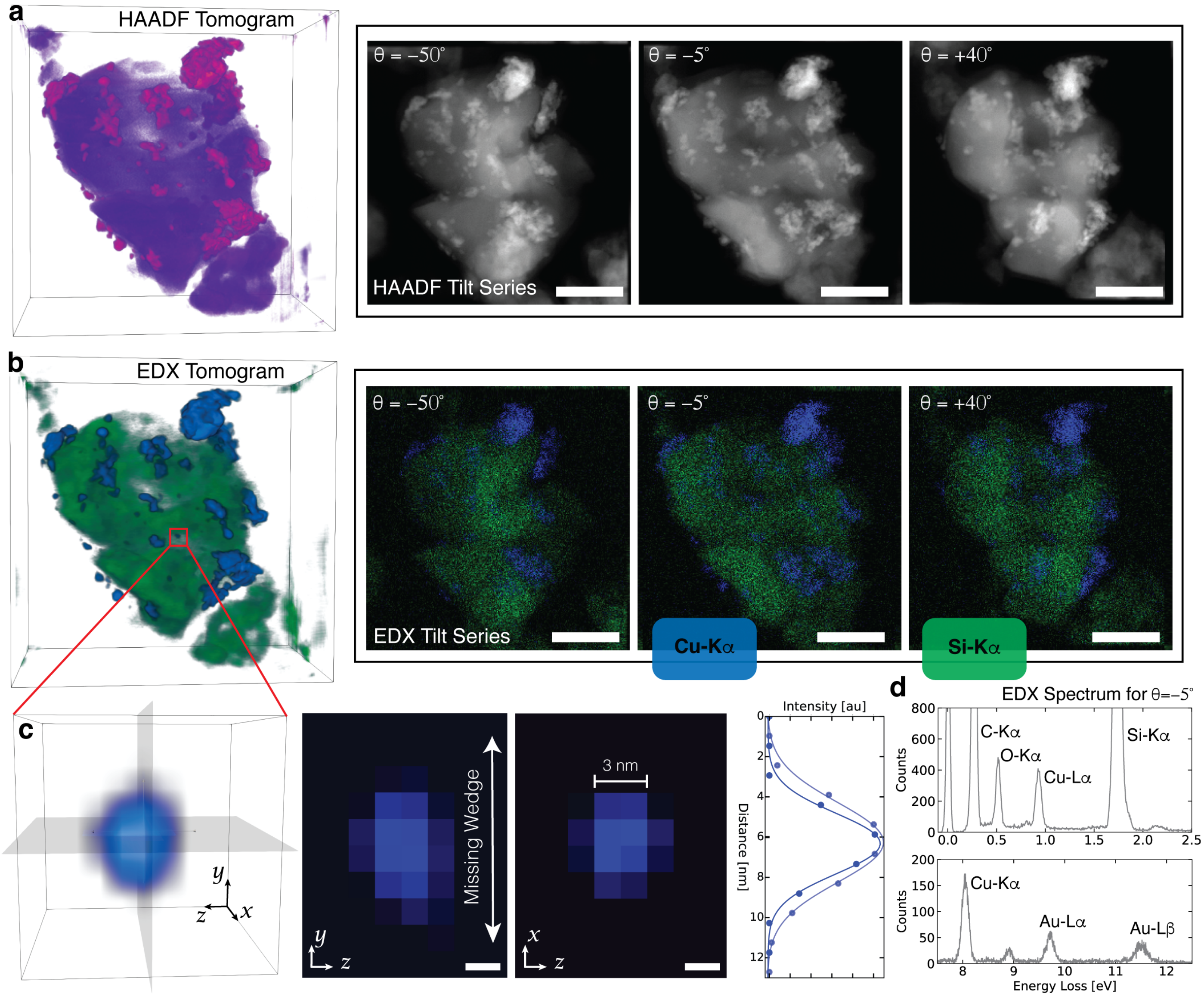

Fig.5 | a Cu-supported silicon carbon (SiC) catalysts designed for the production of fuels and chemicals from biomass. These silica supported catalysts efficiently convert ethanol into acetaldehyde because of their high selectivity and stability [3]. The HAADF reconstruction with a few tilt micrographs is shown on right. **b** The fused multi-modal reconstruction highlighting Cu nanoparticles embedded inside the SiC support and raw EDX maps are shown on the right. Scale bar, 50 nm. **c** The PSF of an individual 3 nm nanoparticle inside the SiC. A few 2D slices of the reconstruction are shown on the right where we see the structure is sharp along the orthogonal axis perpendicular to the missing wedge and approximately a 20% reduction in resolution along the missing wedge direction. Scale bar, 2 nm. **d** EDX spectra for a single tilt.

6 Historical Demonstrations of Multi-Element Chemical Tomography

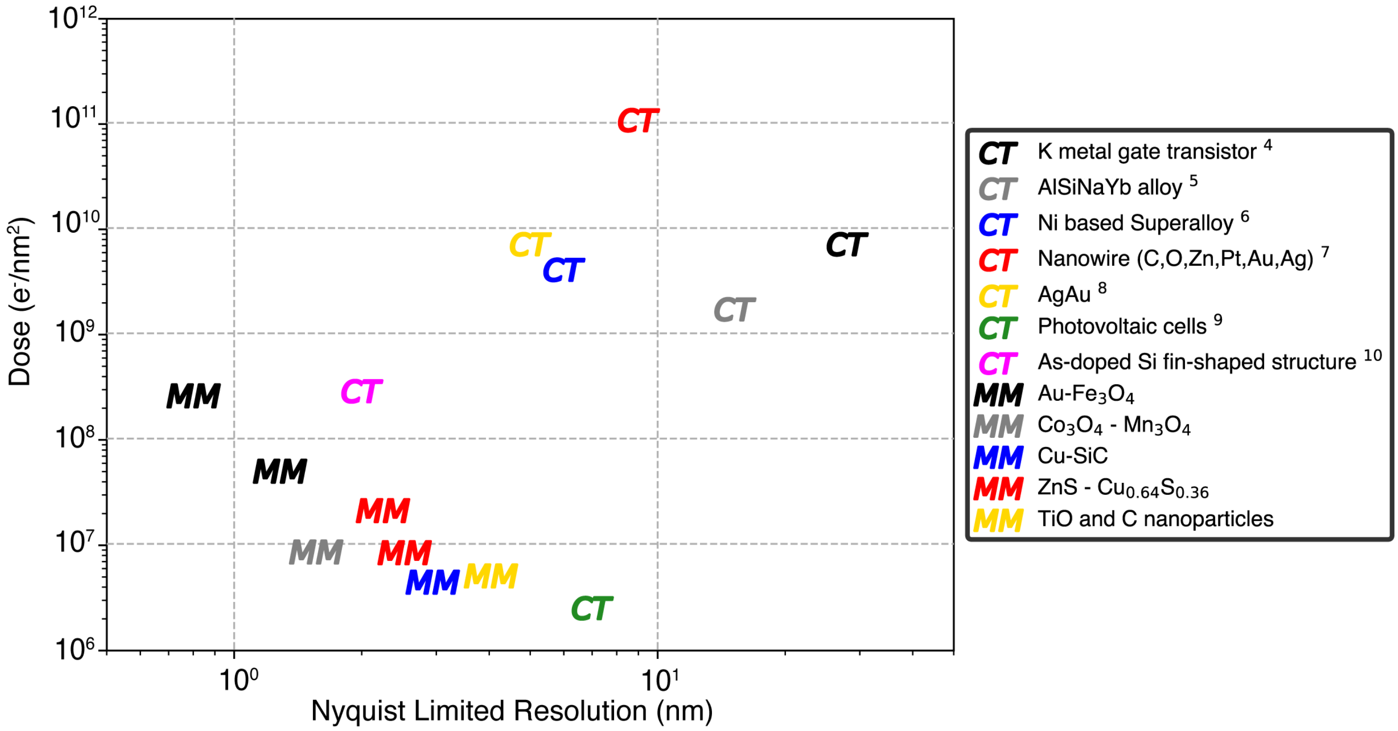

Fig.6 | The reported dose and Nyquist limited resolutions for the fused multi-modal (MM) reconstructions reported in this manuscript are compared to previous multi-element chemical tomography (CT) experiments [4–10]. Note, the actual achieved 3D resolution of previously reported chemical tomography may be lower than the Nyquist resolution.

7 Resolution and Electron Dose for Multi-Modal Electron Tomography

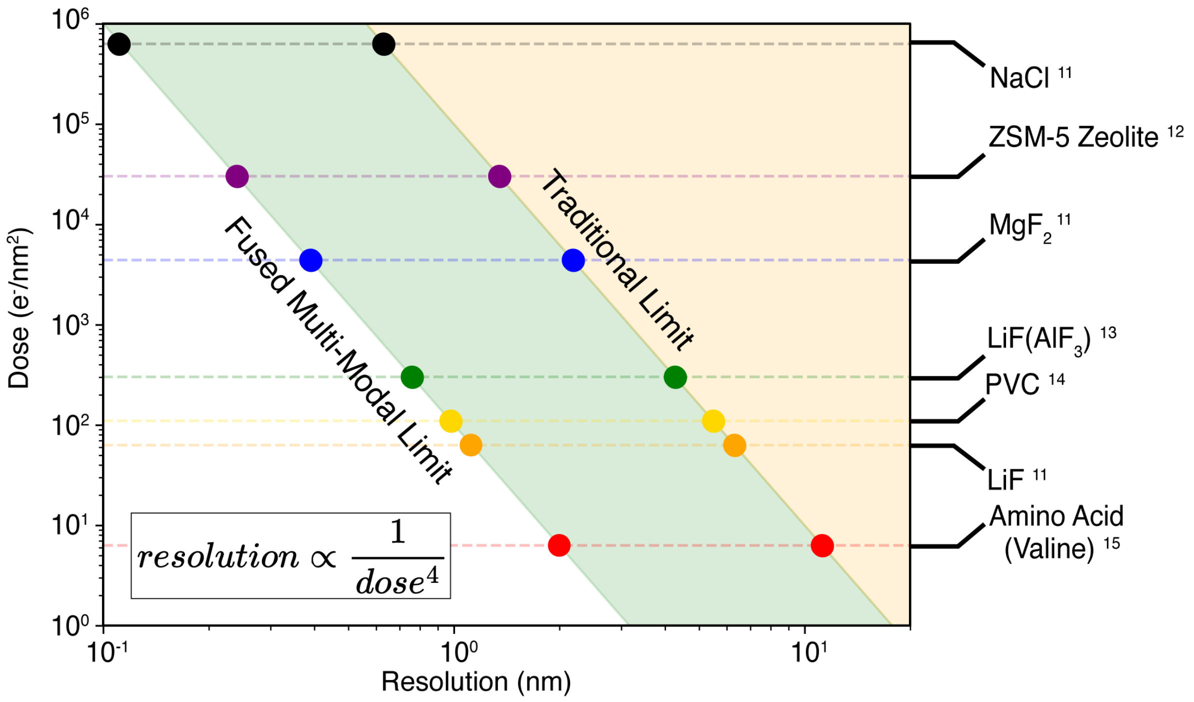

Fig.7 | a Resolution and dose relationship for electron tomography approximates the best achievable resolution for each material [11–15] – assuming an image contrast of 80%[16]. Fused multi-modal electron tomography results in a much higher dose-efficiency which enables higher resolution at any dose limit. **b** Multi-modal (green) and conventional chemical tomography (blue). This relationship between dose and resolution assumes sufficient tomographic sampling is achieved (i.e. many projections)—in practice the actual resolution will be much lower. Dose limited resolution assumes the material is adequately sampled (i.e. Crowther and Nyquist relations)

8 Measuring the Resolution of Au-Fe_3O_4 Nanoparticles in the HAADF

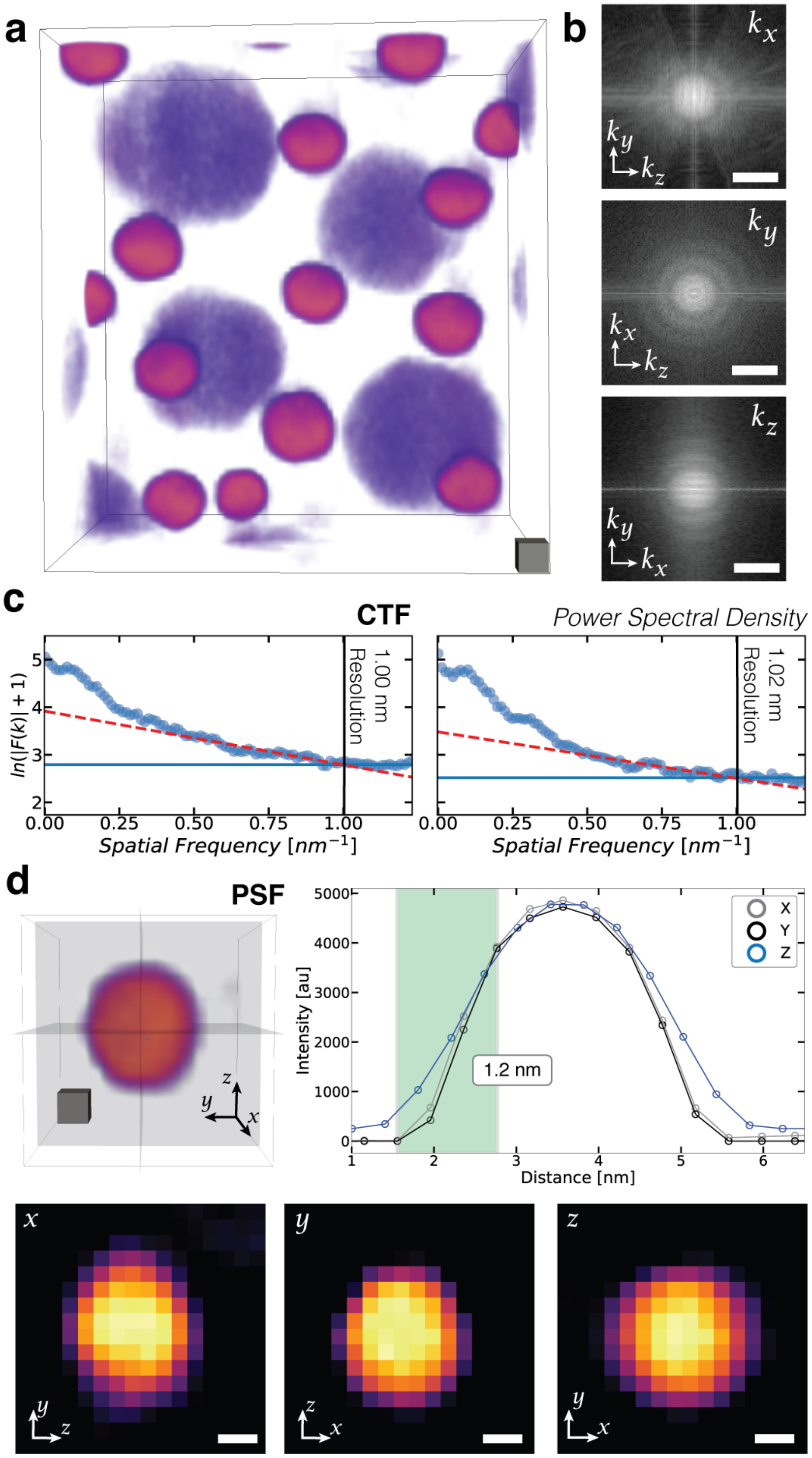

Fig.8 | a Fused EELS tomograms of Au-Fe_3O_4 nanoparticles. Power spectral density of the HAADF reconstruction along the principal axial directions shown on the right. Scale cube, 2 nm^3. Scale bar, 0.5 nm^{-1}. **b** Power spectral density profiles for YZ and XY planes. **c** Line scan profiles of a 2.5 nm Au nanoparticle give a resolution of 1.00, 1.03, and 1.01 nm along the x, y, and z directions.

9 Sampling Requirements of Multi-Modal Tomography on CoO/CuO Nanocubes

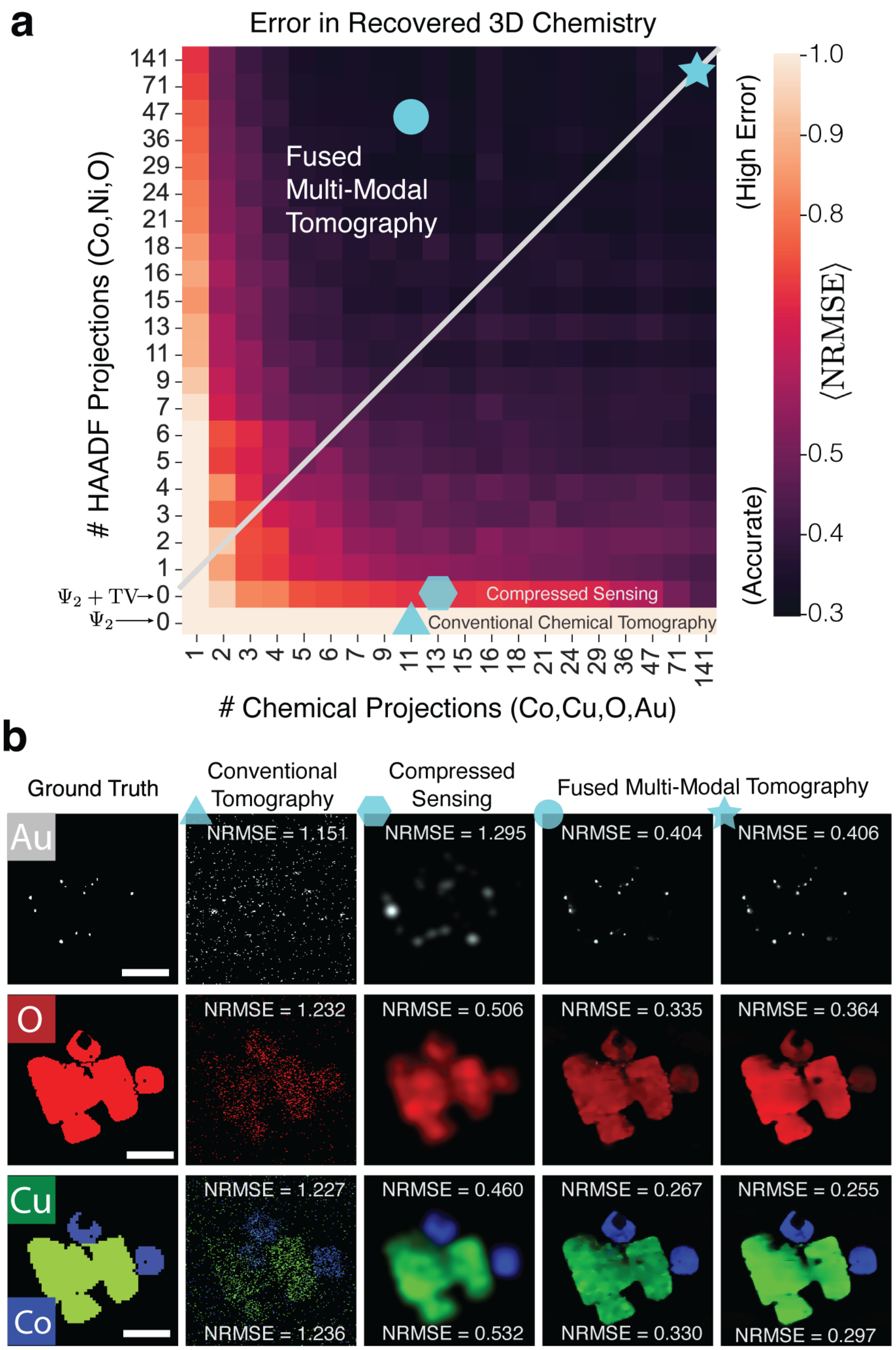

Fig.9 | a An NRMSE map representing reconstruction error as a function of the number of HAADF and chemical tilts. The bottom two rows provide results from non-multi-modal algorithms where the HAADF is not included in the reconstruction process. The last row uses the Poisson Maximum Likelihood term to reconstruct the individual chemical distributions. The second to last row adapts the compressed sensing framework into the reconstruction process by including total variation minimization – thus providing a 2-3 fold reduction in average error. **b** Visualization of four points in the phase diagram corresponding to conventional chemical tomography, regularized tomography (compressed sensing), and low or high-dose fused multi-modal electron tomography. The 3D models were then rendered and colored in Tomviz [17]. Scale bar, 75 nm.

10 Estimating the Sampling Requirements for a CoNiO Composite

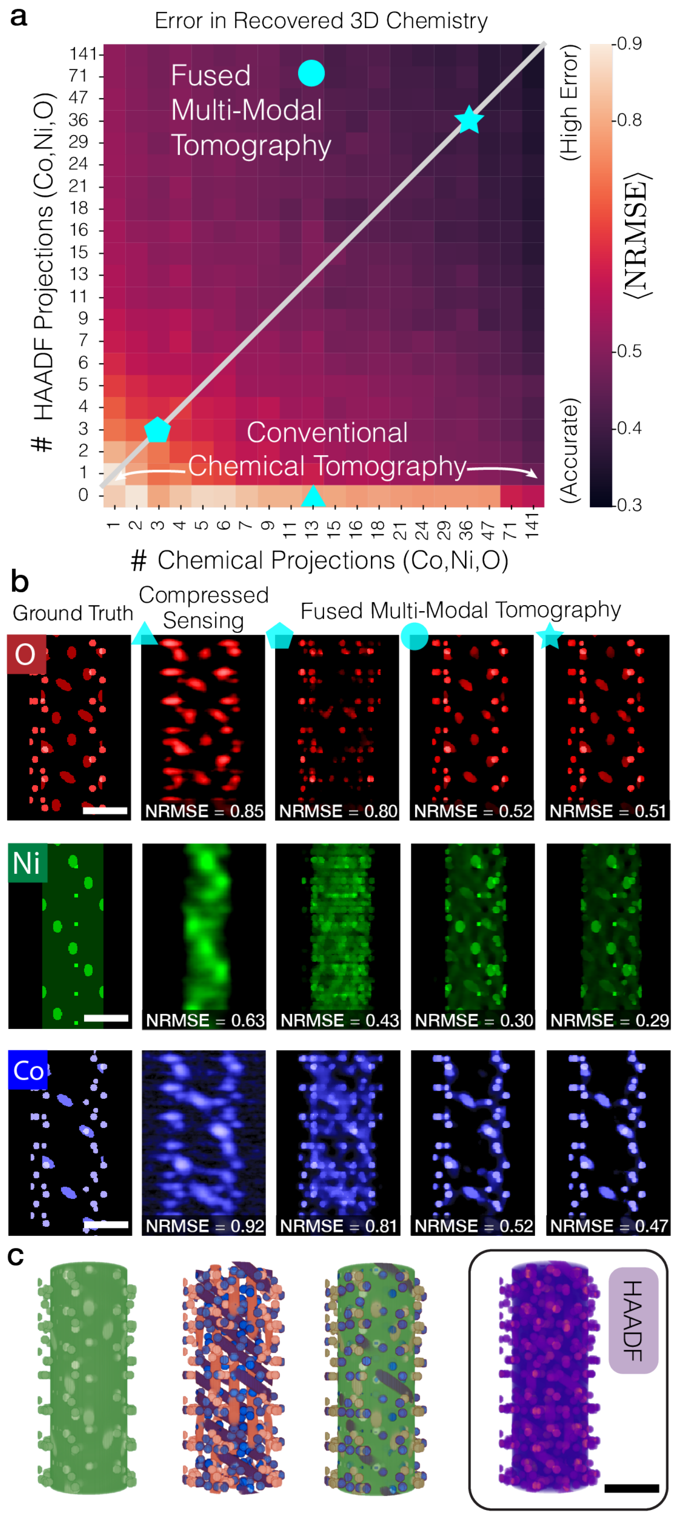

Fig.10 | a A normalized root-mean-square-error (NRMSE) heatmap of a fused multi-modal CoNiO nanotube reconstructions as a function of the number of HAADF and chemical tomographic projections. Brighter pixels denote higher levels of error in the reconstruction when compared to the ground truth. The SNR for the Co, Ni, O, and HAADF modalities were 2.66, 6.46, 3.17, and 2156.16 respectively. **b** Visualization of four points in the phase diagram corresponding to conventional chemical tomography, regularized tomography (compressed sensing), and low or high-dose fused multi-modal electron tomography.**c** The synthetic CoNiO nanotube ground truth 3D models generating synthetic chemical and ADF projections. The 3D models were then rendered and colored in Tomviz [17]. Scale bar, 50 nm.

11 Measuring 3D Stoichiometric Concentration for simulated CuO-CoO Nanocubes

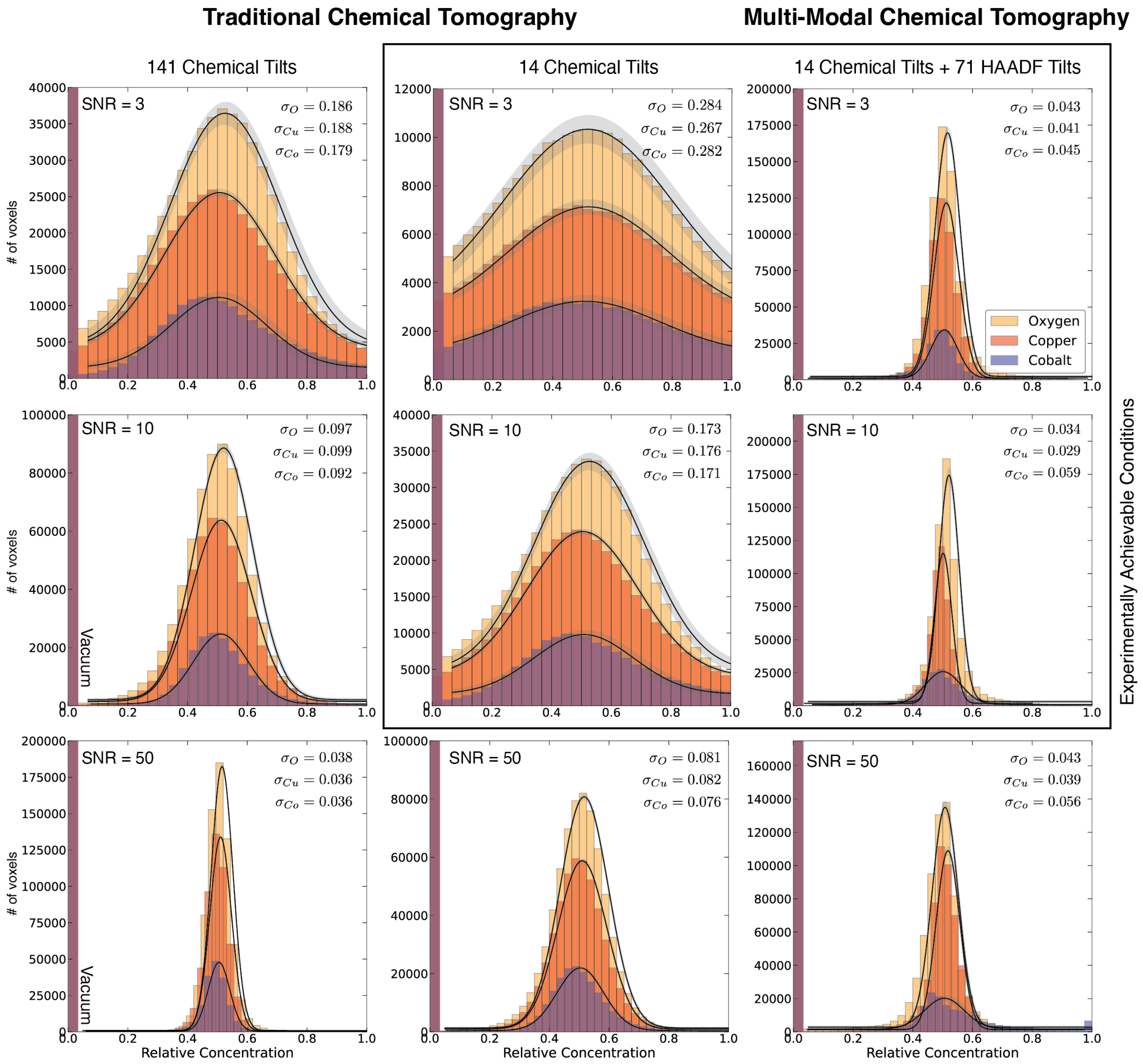

Fig.11 | Histograms of the chemical concentrations for each voxel in the traditional and fused multi-modal tomography reconstructions are shown for the simulated CuO-CoO nanocube system. The mean values of each chemistry are within ± 0.03 of the expected value of 0.5. For traditional chemical tomography, the accuracy improves as SNR increases or more projections are collected. Multi-modal tomography maintains low error, especially for experimentally realistic conditions (e.g. 14 chemical tilts and SNR < 10).

12 Measuring 3D Stoichiometric Concentration of Au-Fe_3O_4 Superlattice Nanoparticles

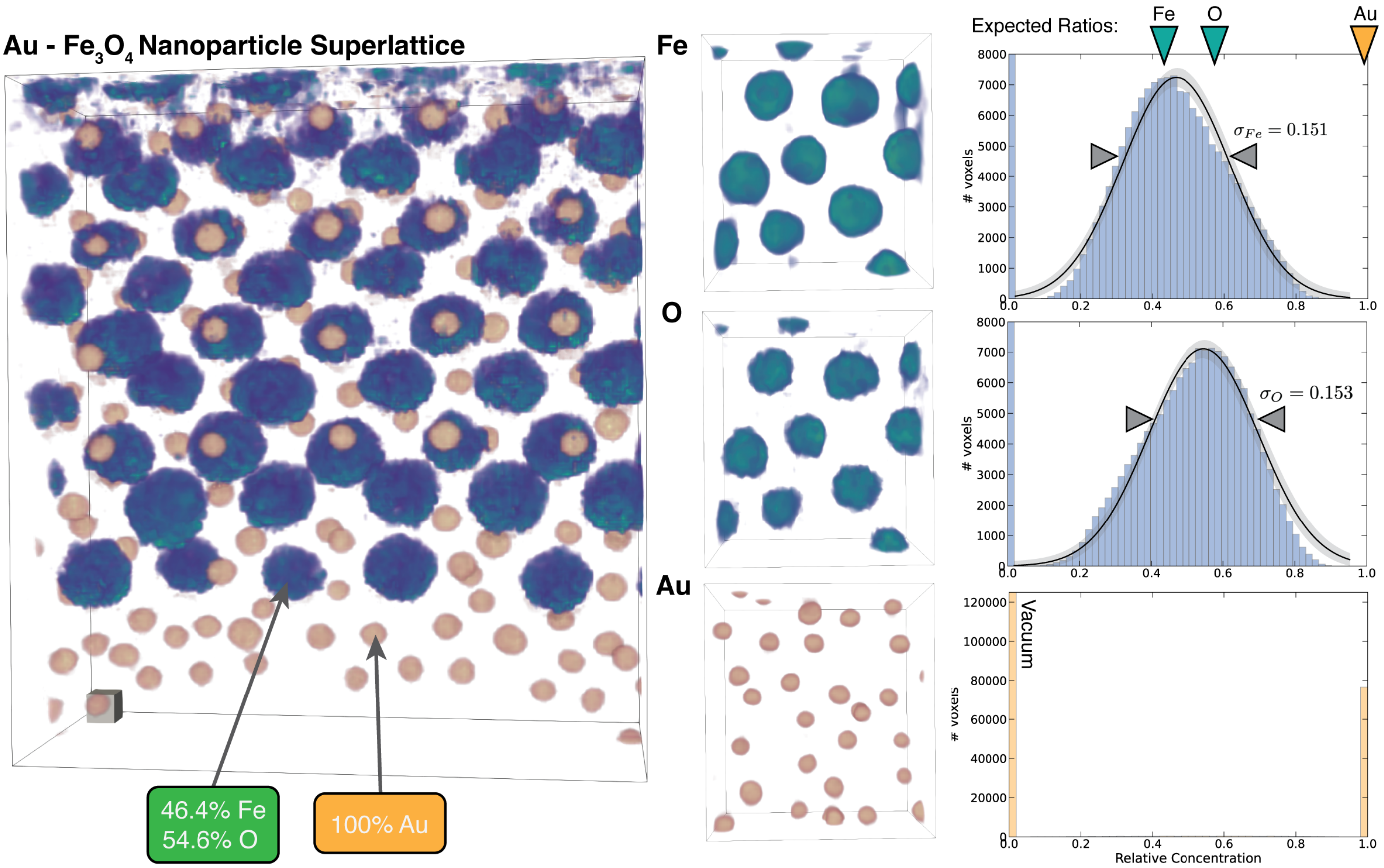

Fig.12 | 3D chemical reconstructions for each element are shown with their corresponding voxel intensity histograms. The mean values and standard deviations are 46.4±15.1%, 54.6±15.3%, 100±0% for Fe, O, and Au, respectively. The expected stochiometry of this system is 42.9%, 57.1%, 100%.

13 Assessing Convergence and Selecting Hyperparameters with Paraeto Front Curves

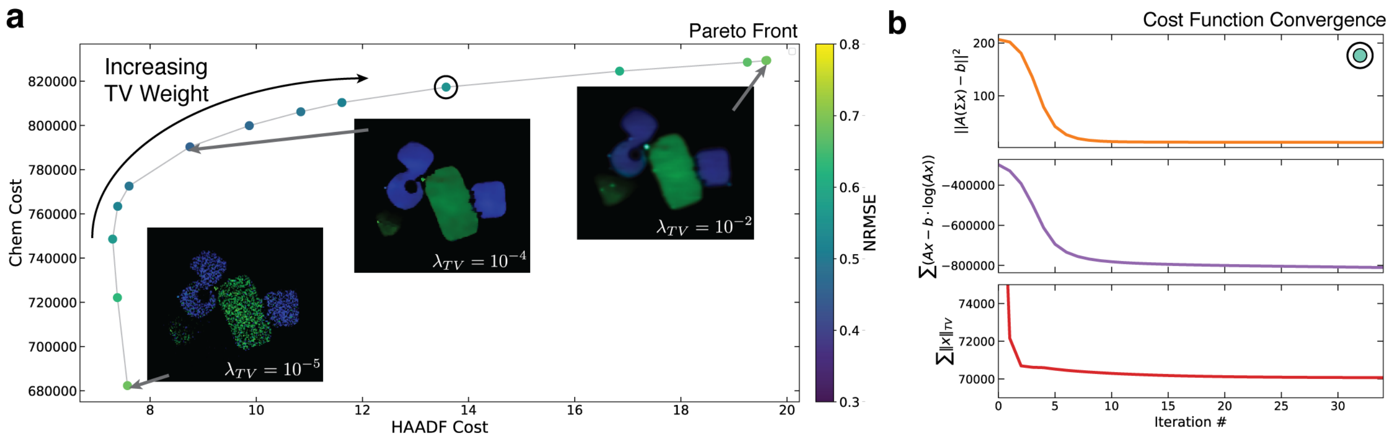

Fig.13 | a Pareto fronts illustrates the relationship between reconstruction quality and regularization parameters for multi-modal electron tomography. Depicted are the tradeoffs from three reconstruction evaluation metrics: the multi-modal, self-consistency and average NRMSE across all elements. We see the highest quality reconstruction (lowest NRMSE) occurs around the inflection point of the pareto front. **b** The three individual components in the cost function plotted throughout the multi-modal electron tomography reconstruction process illustrates smooth asymptotic convergence. Convergence should be confirmed for accurate reconstruction.

14 Consequence of Reconstructing Thick 280 nm Nanoparticles with EELS Tomography

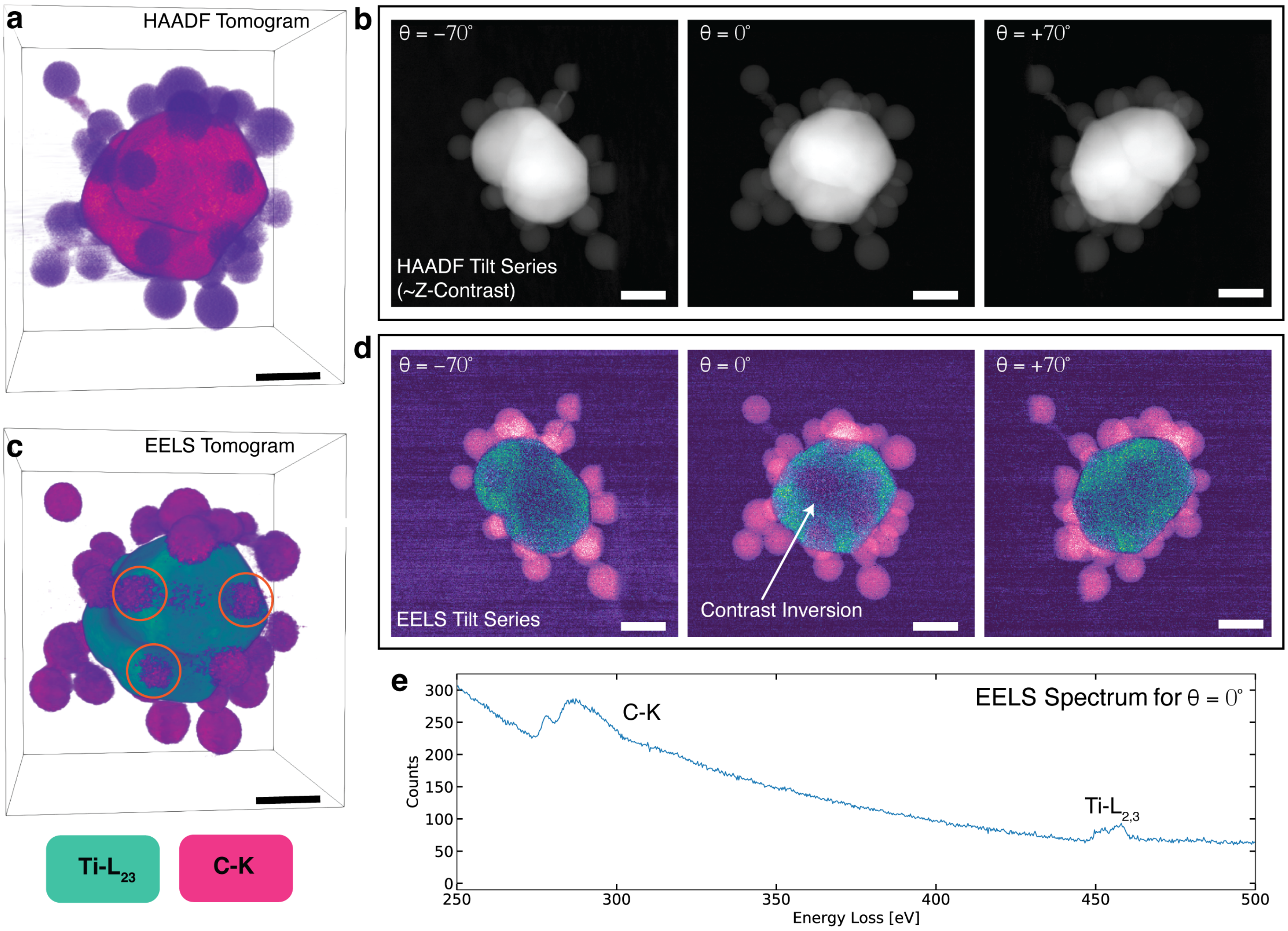

Fig.14 | a The HAADF reconstruction with a few HAADF projections from the tilt series shown on right. These projections were square rooted to visually see the TiO and C nanoparticles. **b** The fused multi-modal reconstruction illustrating TiO nanoparticle decorated by C support with raw EELS maps shown on the right. Scale bar, 100 nm.

The simultaneously acquired HAADF and EELS tilt series for the specimen were collected on a Talos F200X G2 (Thermo Fisher) operated at 200 keV with a probe semi-angle of roughly 10.5 mrad and inner collection semi-angle of 50 mrad. The HAADF projections were collected from -70° to +70° with a +2° angular increment using a Model 2021 Fischione Analytical Tomography Holder. At each tilt angle, a STEM image with a 32 μs dwell time at each pixel of a lateral dimension of 2.015 nm. Simultaneously acquired HAADF and EELS spectrums were acquired at acquired with a 10° angular increment with a dwell time of 2 ms receiving a total electron dose of 4.96×10^4 $e/$Å^2 $(3.48 \times 10^3, 4.61 \times 10^4$ for the HAADF and EELS modality, respectively)

.

15 Raw HAADF Tilt Series for the Au-Fe_3O_4 Nanoparticles

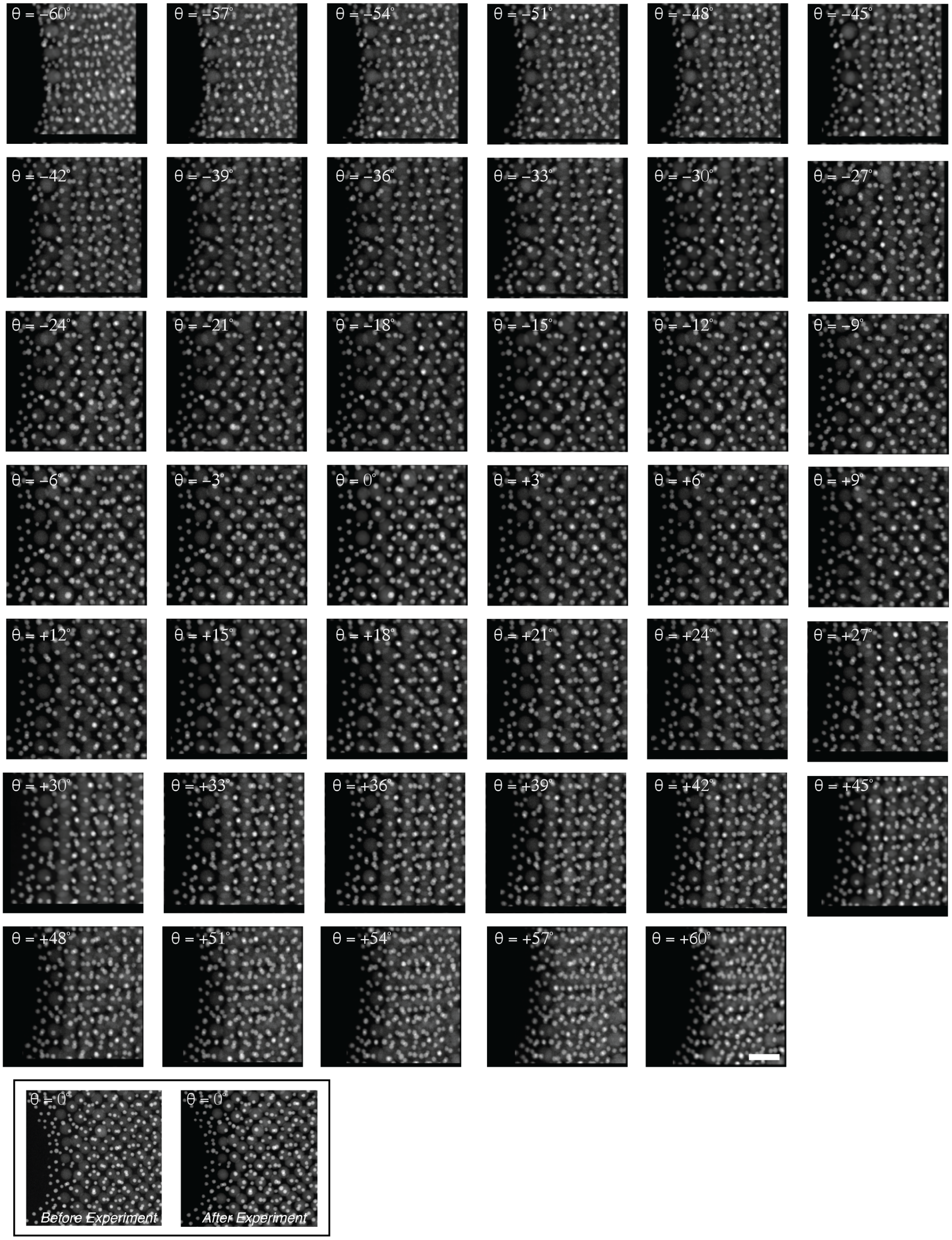

Fig.15 | The 45 projection images with a tilt range from -60° to +60° (shown at the top left of each panel) were measured using ADF-STEM. The total electron dose of the tilt series is 1.72×10^4 e/Å^2. Scale bar shown at the bottom right, 25 nm.

16 Raw EELS Tilt Series for the Au-Fe_3O_4 Nanoparticles

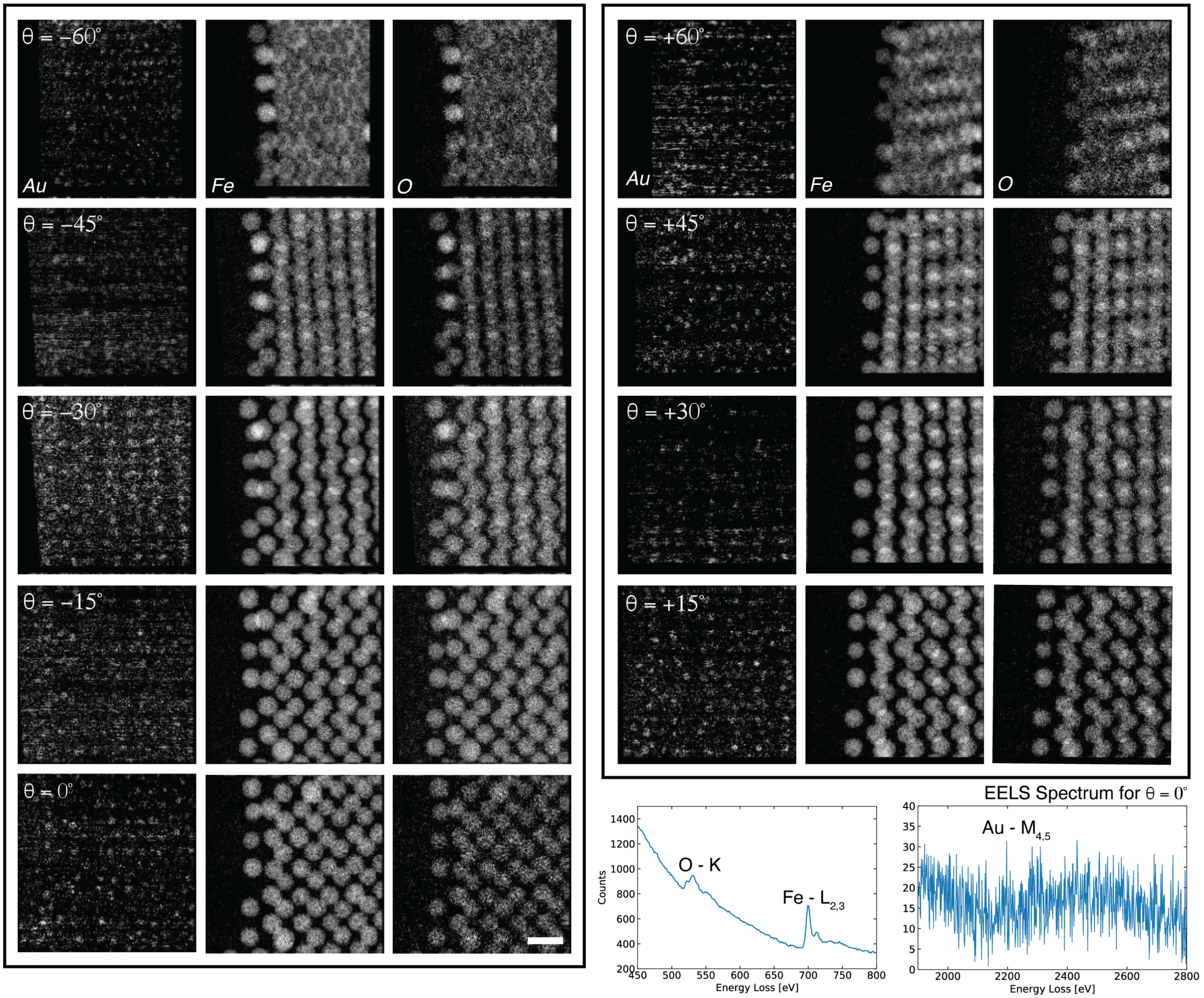

Fig.16 | The 9 EELS maps with a tilt range from -60° to +60° (shown at the top left of each map) were measured using EELS spectroscopy. The total electron dose of the tilt series is 4.73×10^5 e/Å^2. Scale bar is shown at the bottom right, 50 nm.

17 Raw HAADF Tilt Series for the Co_3O_4 - Mn_3O_4 Core-Shell Nanoparticles

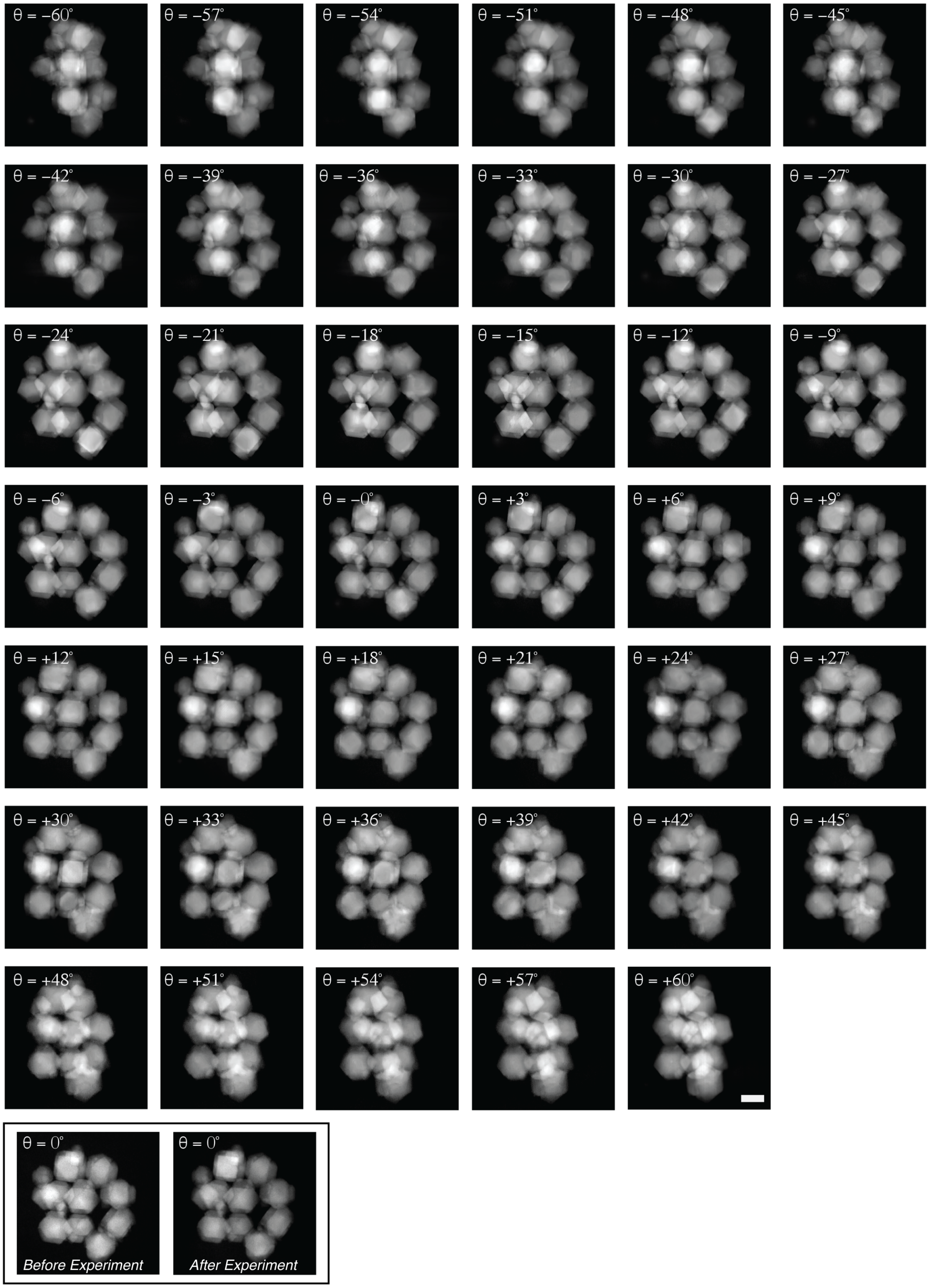

Fig.17 | The 45 projection images with a tilt range from -60° to +60° (shown at the top left of each panel) were measured using ADF-STEM. The total electron dose of the tilt series is 1.16×10^4 e/Å^2. Scale bar is shown at the bottom right, 50 nm.

18 Raw EELS Tilt Series for the Co_3O_4 - Mn_3O_4 Core-Shell Nanoparticles

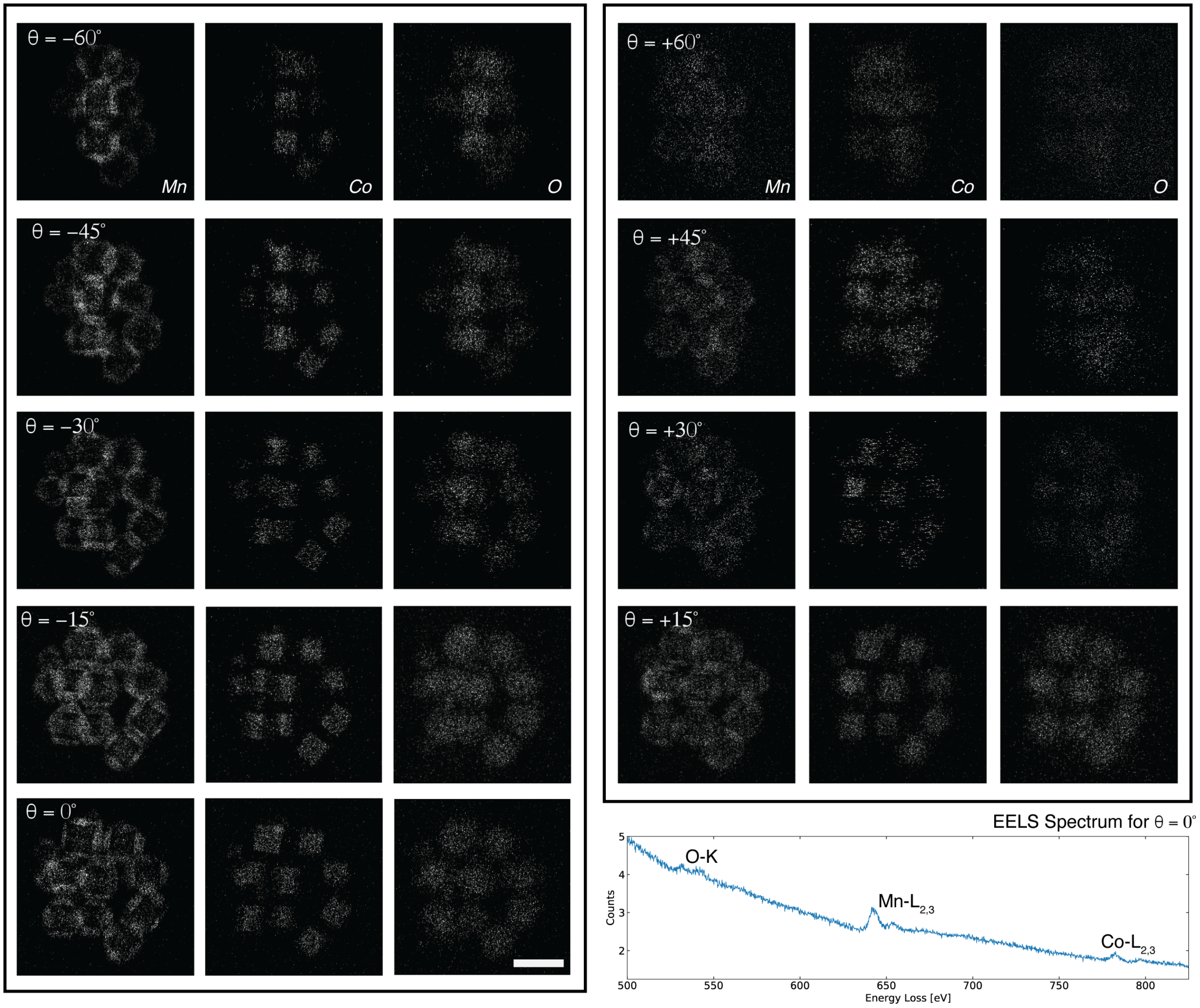

Fig.18 | The 9 EELS maps with a tilt range from -60° to +60° (shown at the top left of each map) were measured using EELS spectroscopy. The total electron dose of the tilt series is 7.21×10^4 e/Å^2. Scale bar is shown at the bottom right, 50 nm.

19 Orthogonal Views of Au - Fe_3O_4 and Co_3O_4 - Mn_3O_4 Nanoparticles

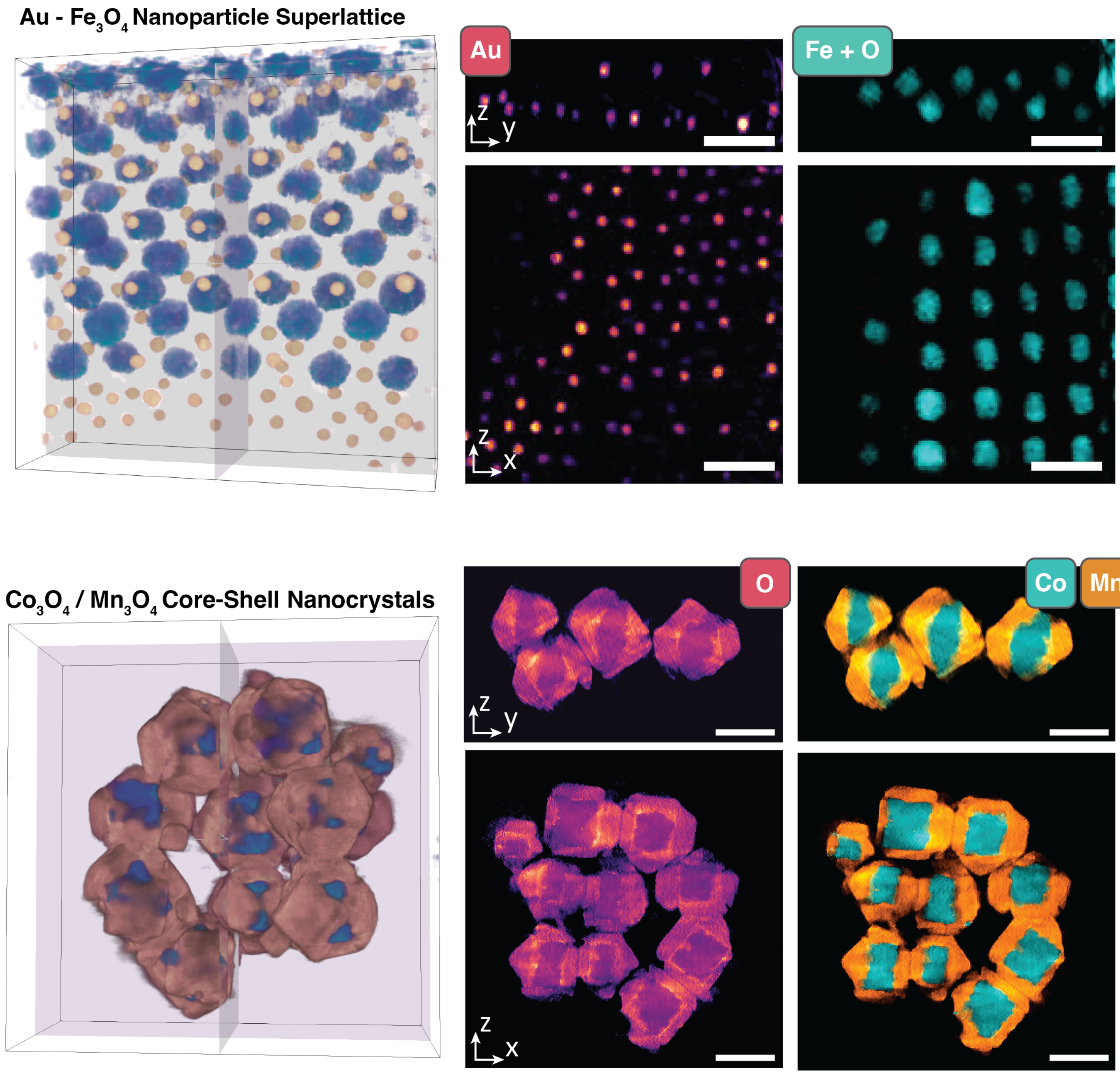

Fig.19 | 3D renderings of the recovered fused chemistries for the Au-Fe_3O_4 nanoparticles (top row) and Co_3O_4 - Mn_3O_4 nanoparticles (bottom row) with the *yz* and *xz* plane cut-view images displayed alongside the tomograms. Scale bars, 25 nm and 50 nm respectively.

20 Drift Correction of Spectrum Maps

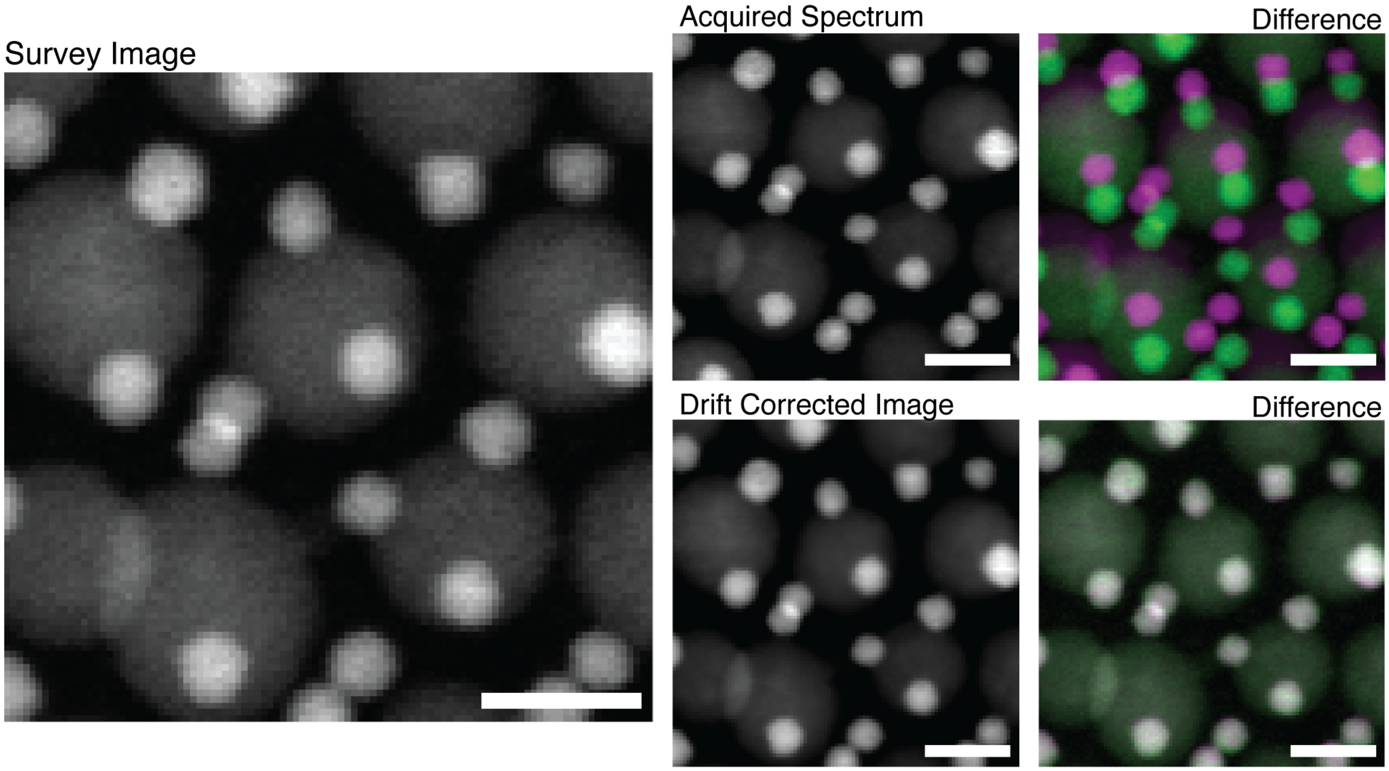

Fig.20 | Drift correction of the spectrum images: a survey image I(x,y), recorded before the spectrum image acquisition, is used as a reference to calculate the affine transformation on the acquired spectrum map. These parameters are subsequently used to correct for drift in the acquired image and all associated spectroscopic signals.

21 SNR Dependency for Successful Fused Multi-Modal Recovery

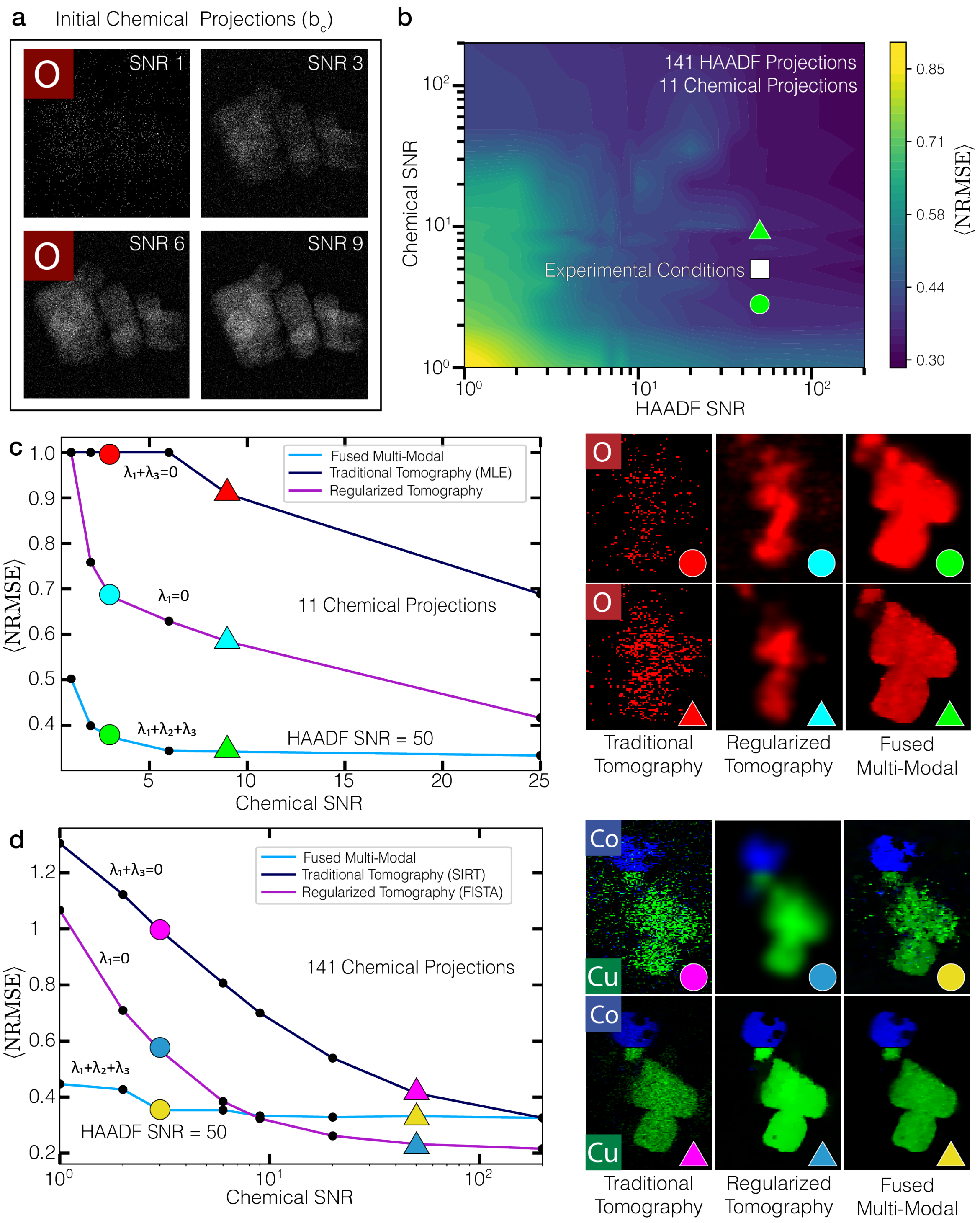

Fig.21 | a The initial corrupted chemical distributions for oxygen in the CuO-CoO synthetic dataset with increasing SNR. We see as we exceed the Rose criterion (SNR $\simeq 5$) internal pores and fine features are visible. **b** A heat map expressing the relationship between average reconstruction error and SNR for either modality (HAADF or Chemistry) when 11 chemical maps ($\Delta\theta = +12^{\circ}$) and 141 HAADF projections ($\Delta\theta = +1^{\circ}$) are available. **c** SNR plot highlighting the average NRMSE as a function of chemical SNR for reconstructions without any regularization or fusion (traditional tomography), without data-fusion (regularized tomography) and within the multi-modal framework. We see substantial improvements in reconstruction quality as we incorporate more terms in the cost function. Most notably, data fusion converges quite rapidly when the SNR is above 6. Visualization of the oxygen elemental reconstruction of the 2D slices from the reconstructions from each of the three curves. **d** SNR plot highlighting the average NRMSE as a function of chemical SNR for traditional tomography with the SIRT reconstruction algorithm, without data-fusion with the FISTA reconstruction algorithm and within the multi-modal framework [18, 19]. When the sampling between the chemistry and HAADF is equivalent and chemical SNR is larger, regularized tomography outperforms mutli-modal tomography. Visualization of the cobalt and copper elemental reconstruction of the 2D slices from the reconstructions from each of the three curves.

Hyperparameter Estimation with Bayesian Optimization for the CuO-CoO Nanocubes

$$\underset{\boldsymbol{x}_i\geq 0}{\arg\min}\ \frac{\lambda_1}{2}\left\|\boldsymbol{A}_h\sum_i(Z_i\boldsymbol{x}_i)^{\gamma}-\boldsymbol{b}_H\right\|_2^2+\lambda_2\sum_i\left(\mathbf{1}^T\boldsymbol{A}_c\boldsymbol{x}_i-\boldsymbol{b}_i^T\log(\boldsymbol{A}_c\boldsymbol{x}_i+\varepsilon)\right)+\lambda_3\sum_i\|\boldsymbol{x}_i\|_{\mathrm{TV}}$$

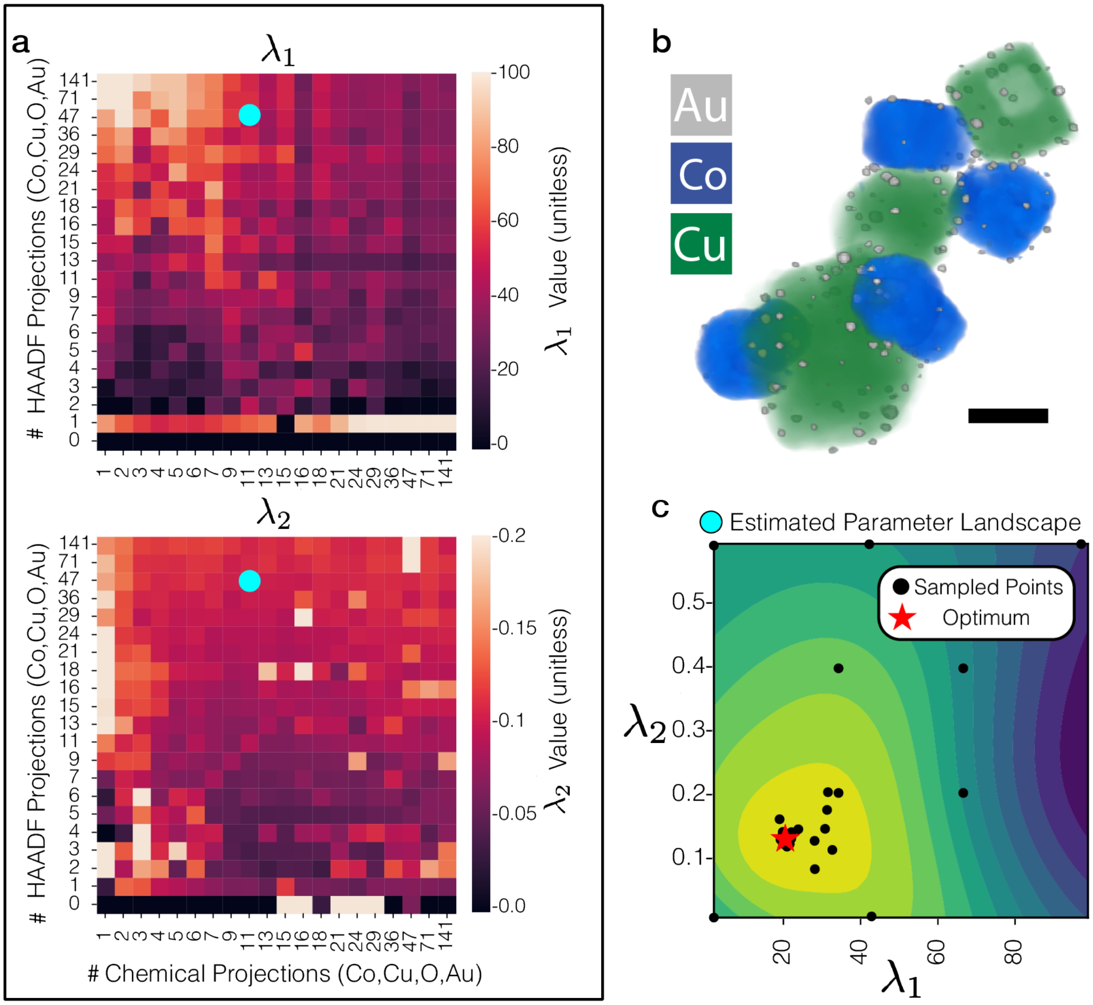

Fig.22 | a Bayesian optimization optimizes the data fusion cost function (shown above) when provided a given number of chemical and HAADF tilts. We see the weights between HAADF and chemical modality (λ_1 and λ_2 respectively) can vary depending on our certainty in either modality. When the number of tilts is low, the corresponding λ value would decrease and vice-versa. Overall, these maps can guide future scientists to produce multi-modal reconstructions with reasonable hyperparameter selections. **b** 3D visualization of the ground truth Au decorated CuO/CoO nanocubes. Scale bar, 75 nm. **c** Bayesian optimization parameter selection landscape where each black dot represents one of the many attempts to find the minimum NRMSE.

23 Hyperparameter Estimation with Bayesian Optimization for CoNiO Composite

$$\arg\min_{\boldsymbol{x}_i \geq 0} \frac{\lambda_1}{2}\left\|\boldsymbol{A}_h \sum_i (Z_i \boldsymbol{x}_i)^\gamma - \boldsymbol{b}_H\right\|_2^2 + \lambda_2 \sum_i \left(\boldsymbol{1}^T \boldsymbol{A}_c \boldsymbol{x}_i - \boldsymbol{b}_i^T \log(\boldsymbol{A}_c \boldsymbol{x}_i + \varepsilon)\right) + \lambda_3 \sum_i \|\boldsymbol{x}_i\|_{\mathrm{TV}}$$

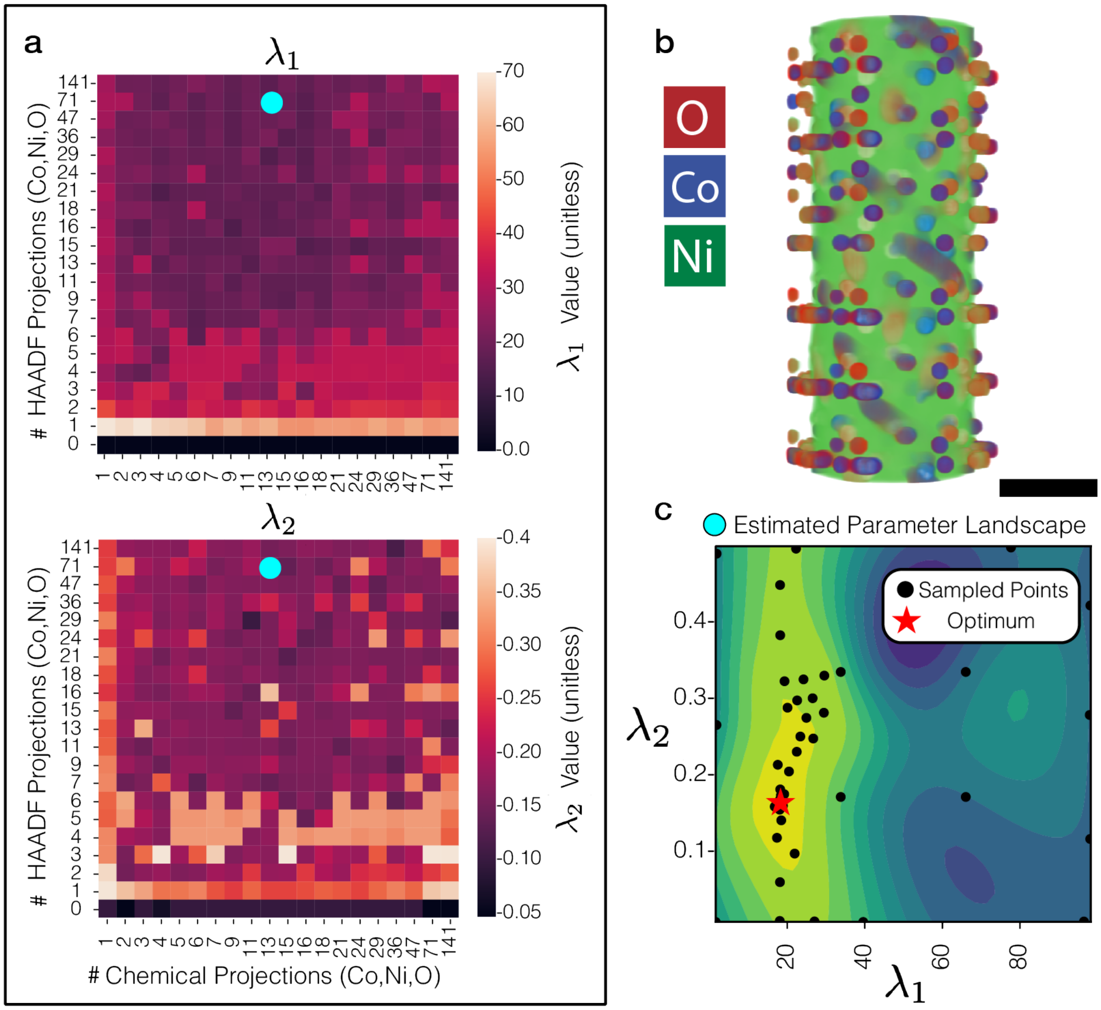

Fig.23 | a Bayesian optimization optimizes the data fusion cost function (shown above) when provided a given number of chemical and HAADF tilts. We see the weights between HAADF and chemical modality (λ_1 and λ_2 respectively) can vary depending on our certainty in either modality. When the number of tilts is low, the corresponding λ value would decrease and vice-versa. Overall, these maps can guide future scientists to produce multi-modal reconstructions with reasonable hyperparameter selections. **b** 3D visualization of the ground truth CoNiO composite structure. Scale bar, 75 nm. **c** Bayesian optimization parameter selection landscape where each black dot represents one of the many attempts to find the minimum NRMSE.

24 Fusion Multi-Modal Electron Tomography Pseudo Code

Algorithm 1 Fused Multi-Modal 3D Chemical Tomography

Objective Function: $\Psi(\boldsymbol{x}) = \frac{\lambda_1}{2}\left\|\boldsymbol{A}_h \sum_i (Z_i \boldsymbol{x}_i)^\gamma - \boldsymbol{b}_h\right\|_2^2 + \lambda_2 \sum_i \left(\mathbf{1}^T \boldsymbol{A}_c \boldsymbol{x}_i - \boldsymbol{b}_i^T \log(\boldsymbol{A}_c \boldsymbol{x}_i + \varepsilon)\right) + \lambda_{\mathrm{TV}} \sum_i \|\boldsymbol{x}_i\|_{\mathrm{TV}}$

Input: $\boldsymbol{b}_h \in \mathbb{R}^{n_y \cdot N^{\mathrm{proj}}_{\mathrm{HAADF}} \times n_x}$ ▷ HAADF Tilt Series

Input: $\boldsymbol{b}_c \in \mathbb{R}^{n_i \cdot n_y \cdot N^{\mathrm{proj}}_{\mathrm{chem}} \times n_x}$ ▷ Chemical Tilt Series

Output: $\boldsymbol{x} \in \mathbb{R}^{n_i \cdot n_y \cdot n_y \times n_x}$ ▷ Reconstructed Chemical Tomogram

$N_{\text{iter}} = 100, \varepsilon = 0.1$

$\boldsymbol{x}_i^0 = \arg\min_{\boldsymbol{x}} \Psi_2(\mathbf{0})$ ▷ Initialize first iterate with reconstruction from raw chemical maps

for $k = 1, N_{iter}$ **do** ▷ Main Loop

  for $s = 1, n_x$ **do** ▷ Apply the Gradient for Each Slice Along the Axis Parallel to Tilt Axis

    $\boldsymbol{x}_s^k = \boldsymbol{x}_s^{k-1} - (\lambda_1 \nabla_{\boldsymbol{x}} \Psi_1(\boldsymbol{x}_s^{k-1}) + \lambda_2 \nabla_{\boldsymbol{x}} \Psi_2(\boldsymbol{x}_s^{k-1}))$

  end for

  for $i = 1, n_i$ **do**

    $\boldsymbol{x}_i^k = \text{tv\_fgp\_3D}(\boldsymbol{x}_i^k, \lambda_{TV})$ ▷ Channel-Wise TV Minimization

  end for

end for

return $\boldsymbol{x}$

Comments:

$\nabla_{\boldsymbol{x}} \Psi_1(\boldsymbol{x}) = -\gamma \mathrm{diag}\left(\boldsymbol{x}^{\gamma-1}\right) \boldsymbol{\Sigma}^T \boldsymbol{A}_h^T \Big(\boldsymbol{A}_h (\boldsymbol{\Sigma} \boldsymbol{x}^\gamma) - \boldsymbol{b}_h\Big)$

$\boldsymbol{\Sigma} \in \mathbb{R}^{n_y \cdot n_y \times n_y \cdot n_y \cdot n_i}$ expresses the summation operation as matrix vector multiplication.

$\nabla_{\boldsymbol{x}_i} \Psi_2(\boldsymbol{x}_i) = \boldsymbol{A}_c^T \Big((\boldsymbol{A}_c \boldsymbol{x}_i - \boldsymbol{b}_i) \oslash (\boldsymbol{A}_c \boldsymbol{x}_i + \varepsilon)\Big)$

$\oslash$ is element-wise division.

$\boldsymbol{x}_i \in \mathbb{R}^{n_y \cdot n_y \times n_x}$ is the reconstruction for element i.

$\boldsymbol{b}_i \in \mathbb{R}^{n_y \cdot N^{\mathrm{proj}}_{\mathrm{chem}} \times n_x}$ is the chemical tilt series for element i.

$\boldsymbol{x}_s \in \mathbb{R}^{n_y \cdot n_y \cdot n_i}$.

Algorithm 2 3D Gradient Projection (GP) Method (DOI: 10.1109/TIP.2009.2028250)

Input: $\boldsymbol{b} \in \mathbb{R}^{n_x \times n_y} \rightarrow$ 3D Volume, $\lambda \rightarrow$ Regularization Parameter, $ng \rightarrow$ Number of Iterations
Output: $\boldsymbol{x}^* \rightarrow$ Optimal Solution

function TV_GP_3D($\mathbf{x}, \lambda, ng$)
  $\boldsymbol{p}_x^0 = \mathbf{0} \in \mathbb{R}^{(m-1)\times n \times k}, \quad \boldsymbol{p}_y^0 = \mathbf{0} \in \mathbb{R}^{m\times (n-1) \times k}, \quad \boldsymbol{p}_z^0 = \mathbf{0} \in \mathbb{R}^{m\times n \times (k-1)}$
  for $k = 1, ng$ **do** ▷ Main Loop
    $(\boldsymbol{p}_x^k, \boldsymbol{p}_y^k, \boldsymbol{p}_z^k) = P_{\mathcal{P}}\left[(\boldsymbol{p}_x^{k-1}, \boldsymbol{p}_y^{k-1}, \boldsymbol{p}_z^{k-1}) + \frac{1}{26\lambda}\mathcal{L}^T\left(P_C[\boldsymbol{b} - \lambda\mathcal{L}(\boldsymbol{p}_x^{k-1}, \boldsymbol{p}_y^{k-1}, \boldsymbol{p}_z^{k-1})]\right)\right]$
  end for
  return $\boldsymbol{x}^* = P_C[\boldsymbol{b} - \lambda\mathcal{L}(\boldsymbol{p}_x^{ng}, \boldsymbol{p}_y^{ng}, \boldsymbol{p}_z^{k-1}))]$
end function

function $\mathcal{L}(\boldsymbol{p},\boldsymbol{q},\boldsymbol{r})$ ▷ Linear Operation
  for $i = 1, \ldots, n_x$ **do**
    for $j = 1, \ldots, n_y$ **do**
      for $k = 1, \ldots, n_k$ **do**
        if $i == 0$ or $i == n_x$ **do** $p_{i,j,k} = 0$
        if $j == 0$ or $j == n_y$ **do** $q_{i,j,k} = 0$
        if $k == 0$ or $k == n_k$ **do** $r_{i,j,k} = 0$
        $\mathcal{L}(\boldsymbol{p},\boldsymbol{q},\boldsymbol{r})_{i,j,k} = p_{i,j,k} + q_{i,j,k} + r_{i,j,k} - p_{i-1,j,k} - q_{i,j-1,k} - r_{i,j,k-1}$
      end for
    end for
  end for
  return $\mathcal{L}(\boldsymbol{p},\boldsymbol{q},\boldsymbol{r})$
end function

function $\mathcal{L}^T(\boldsymbol{x})$
  for $i = 1, \ldots, n_x - 1$ **do**
    for $j = 1, \ldots, n_y - 1$ **do**
      for $k = 1, \ldots, n_z - 1$ **do**
        $p_{i,j} = x_{i,j} - x_{i+1,j,k}$
        $q_{i,j} = x_{i,j} - x_{i,j+1,k}$
        $r_{i,j} = x_{i,j} - x_{i,j,k+1}$
      end for
    end for
  end for
  return $(\boldsymbol{p},\boldsymbol{q},\boldsymbol{r})$
end function

Algorithm 3 Projection and Proximal Functions for the 3D Gradient Projection Method (DOI: 10.1109/TIP.2009.2028250)

```
function P_C(x)                                   ▷ Orthogonal Projection Operator Onto Convex Set (Non-negativity)
    return max{0, x}
end function

function P_P(p, q, r)                             ▷ Projection Operator for Isotropic TV Norm
    for i = 1, ..., n_x − 1 do
        for j = 1, ..., n_y − 1 do
            for k = 1, ..., n_z − 1 do
                denom = sqrt(p_{i,j,k}^2 + q_{i,j,k}^2 + r_{i,j,k}^2)
                p_{i,j,k} = p_{i,j,k} / {max{1, denom}}
                q_{i,j,k} = q_{i,j,k} / max{1, denom}
                r_{i,j,k} = r_{i,j,k} / max{1, denom}
            end for
        end for
    end for
    return (p, q, r)
end function

function P_P(p, q, r)                             ▷ Projection Operator for Anisotropic TV Norm
    for i = 1, ..., n_x − 1 do
        for j = 1, ..., n_y − 1 do
            for k = 1, ..., n_z − 1 do
                p_{i,j,k} = p_{i,j,k} / max{1, |p_{i,j,k}|}
                q_{i,j,k} = q_{i,j,k} / max{1, |q_{i,j,k}|}
                r_{i,j,k} = r_{i,j,k} / max{1, |r_{i,j,k}|}
            end for
        end for
    end for
    return (p, q, r)
end function
```

Supplementary References

1. Getreuer, P. Rudin-Osher-Fatemi Total Variation Denoising using Split Bregman. *Image Processing On Line* **2,** 74–95. doi:10.5201/ipol.2012.g-tvd (2012).
2. Ha, D.-H. *et al.* Solid-solid phase transformations induced through cation exchange and strain in 2D heterostructured copper sulfide nanocrystals. *Nano Lett.,* 7090–7099. doi:10.1021/nl5035607 (2014).
3. Li, M.-Y., Lu, W.-D., He, L., Schüth, F. & Lu, A.-H. Tailoring the Surface Structure of Silicon Carbide Support for Copper Catalyzed Ethanol Dehydrogenation. *Chem Cat Chem* **11,** 481–487. doi:10.1002/cctc.201801742 (2019).
4. Lepinay, K., Lorut, F., Pantel, R. & Epicer, T. Chemical 3D tomography of 28 nm high K metal gate transistor: STEM XEDS experimental method and results. *Micron* **47,** 43–49. doi:10.1016/j.micron.2013.01.004 (2013).
5. Huber, R., Haberfehlner, G., Holler, M., Kothleitner, G. & Bredies, K. Total generalized variation regularization for multi-modal electron tomography. *Nanoscale* **11,** 5617–5632. doi:10.1039/C8NR09058K (2019).
6. Rossouw, D. *et al.* Blind source separation aided characterization of the γ strengthening phase in an advanced nickel-based superalloy by spectroscopic 4D electron microscopy. *Acta Materialia* **107,** 229–238. doi:10.1016/j.actamat.2016.01.042 (2016).
7. Burdet, P., Saghi, Z., Filippin, A., Borras, A. & Midgley, P. A novel 3D absorption correction method for quantitative EDX-STEM tomography. *Ultramicroscopy* **160,** 118–129. doi:10.1016/j.ultramic.2015.09.012 (2016).
8. Slater, T. *et al.* STEM-EDX tomography of bimetallic nanoparticles: A methodological investigation. *Ultramicroscopy* **162,** 61–73. doi:10.1016/j.ultramic.2015.10.007 (2016).
9. Pfannmoller, M. *et al.* Quantitative Tomography of Organic Photovoltaic Blends at the Nanoscale. *Nano Lett.* **15,** 6634–6642. doi:10.1021/acs.nanolett.5b02437 (2015).
10. Jacob, M. *et al.* Correlative STEM-HAADF and STEM-EDX tomography for the 3D morphological and chemical analysis of semiconductor devices. *Semicond. Sci. Technol.* **36,** 035006. doi:10.1088/1361-6641/abd925 (2021).
11. Muray, A., Scheinfein, M., Isaacson, M. & Adesida, I. Radiolysis and resolution limits of inorganic halide resists. *Journal of Vacuum Science and Technology B: Microelectronics Processing and Phenomena* **3,** 367–372. doi:10.1116/1.583265 (1985).
12. Pan, M. & Crozier, P. Quantitative imaging and diffraction of zeolites using a slow-scan CCD camera. *Ultramicroscopy* **52,** 487–498. doi:10.1016/0304-3991(93)90065-6 (1993).
13. Langheinrich, W. & Beneking, H. The resolution of the inorganic electron beam resist LiF(AlF3). *Microelectron. Eng.* **23,** 287–290. doi:10.1016/0167-9317(94)90157-0 (1994).
14. Vesely, D. Electron beam damage of amorphous synthetic polymers. *Ultramicroscopy* **14,** 279–290. doi:10.1016/0304-3991(84)90096-2 (1984).
15. Cosslett, V. Radiation damage in the high resolution electron microscopy of biological materials: A review. *Journal of Microscopy* **113,** 113–129. doi:10.1111/j.1365-2818.1978.tb02454.x (1978).
16. McEwen, B. F., Marko, M., Hsieh, C.-E. & Mannella, C. Use of frozen-hydrated axonemes to assess imaging parameters and resolution limits in cryoelectron tomography. *J. Struct. Biol.* **138,** 47–57. doi:10.1016/S1047-8477(02)00020-5 (2002).
17. Schwartz, J. *et al.* Real-time 3D analysis during electron tomography using tomviz. *Nat. Comm.* **2022,** 4458. doi:10.1038/s41467-022-32046-0 (2022).
18. Gilbert, P. Iterative Methods for the Three-Dimensional Reconstruction of an Object from Projections. *J. Theor. Biol.* **36,** 105–117. doi:10.1016/0022-5193(72)90180-4 (1972).
19. Beck, A. & Teboulle, M. A Fast Iterative Shrinkaage-Thresholding Algorithm for Linear Inverse Problems. *SIAM J. Imaging Sciences* **2,** 183–202. doi:10.1137/080716542 (2009).
